# Regulatory functional territory of PLK-1 and their substrates beyond mitosis

**DOI:** 10.18632/oncotarget.16290

**Published:** 2017-03-16

**Authors:** Shiv Kumar, Garima Sharma, Chiranjib Chakraborty, Ashish Ranjan Sharma, Jaebong Kim

**Affiliations:** ^1^ Department of Biochemistry, Institute of Cell Differentiation and Aging, Hallym University, College of Medicine, Chucheonsi, Gangwondo, Republic of Korea; ^2^ Institute For Skeletal Aging & Orthopedic Surgery, Hallym University, College of Medicine, Chucheonsi, Gangwondo, Republic of Korea; ^3^ Department of Bio-informatics, School of Computer and Information Sciences, Galgotias University, Greater Noida, Uttar Pradesh, India

**Keywords:** polo-like kinase-1, transcription and translation, ciliogenesis, checkpoint adaptation and recovery, DNA damage response

## Abstract

Polo-like kinase 1 (PLK-1) is a well-known (Ser/Thr) mitotic protein kinase and is considered as a proto-oncogene. As hyper-activation of PLK-1 is broadly associated with poor prognosis and cancer progression, it is one of the most extensively studied mitotic kinases. During mitosis, PLK-1 regulates various cell cycle events, such as spindle pole maturation, chromosome segregation and cytokinesis. However, studies have demonstrated that the role of PLK-1 is not only restricted to mitosis, but PLK-1 can also regulate other vital events beyond mitosis, including transcription, translation, ciliogenesis, checkpoint adaptation and recovery, apoptosis, chromosomes dynamics etc. Recent reviews have tried to define the regulatory role of PLK-1 during mitosis progression and tumorigenesis, but its’ functional role beyond mitosis is still largely unexplored. PLK-1 can regulate the activity of many proteins that work outside of its conventional territory. The dysregulation of these proteins can cause diseases such as Alzheimer’s disease, tumorigenesis etc. and may also lead to drug resistance. Thus, in this review, we discussed the versatile role of PLK-1 and tried to collect data to validate its’ functional role in cell cycle regulation apart from mitosis.

## INTRODUCTION

Polo-like kinase-1 (PLK-1) is a well-known mitotic kinase and is a widely studied member of the PLK family [1]. The presence of PLK-1 was first reported in *Drosophila melanogaster* as a mitotic regulator of the cell cycle [2]. Since then, the functions of PLK-1 have been extensively studied in mitosis during cell division [3, 4]. PLK-1 is a Ser/Thr protein kinase that is greatly conserved in wide range of eukaryotes. The PLK family members are recognized by different names among all species, including human PLK-1-5, *Xenopus laevis* (Plx1-3), *Schizosaccharomyces pombe* (Plo1), *Caenorhabditis elegance* (Plc1), and *Saccharomyces cerevisiae* (Cdc5). Among the human kinome, the PLK family members constitute an important phylogenetic space. All of the PLK family members have highly conserved domains. Specifically, the N-terminal catalytic kinase domain (KD) and two C-terminal regulatory domains, which are well-known as a polo-box domain (PBD), are highly conserved. Interestingly, PLK-4 functions with only one regulatory PBD domain, while PLK-5 does not contain any kinase domains [5–7]. Many studies have tried to delineate the precise and proper regulatory mechanisms of PLK-1 in mitosis, specially during the spindle pole maturation (SPM), metaphase-anaphase transition, chromosome segregation, cytokinesis, and spindle assembly checkpoint (SAC), because deregulation of these key events during mitosis are responsible for the onset of tumorigenesis as well as the poor prognosis of cancer [3, 8–12].

**Figure 1:**
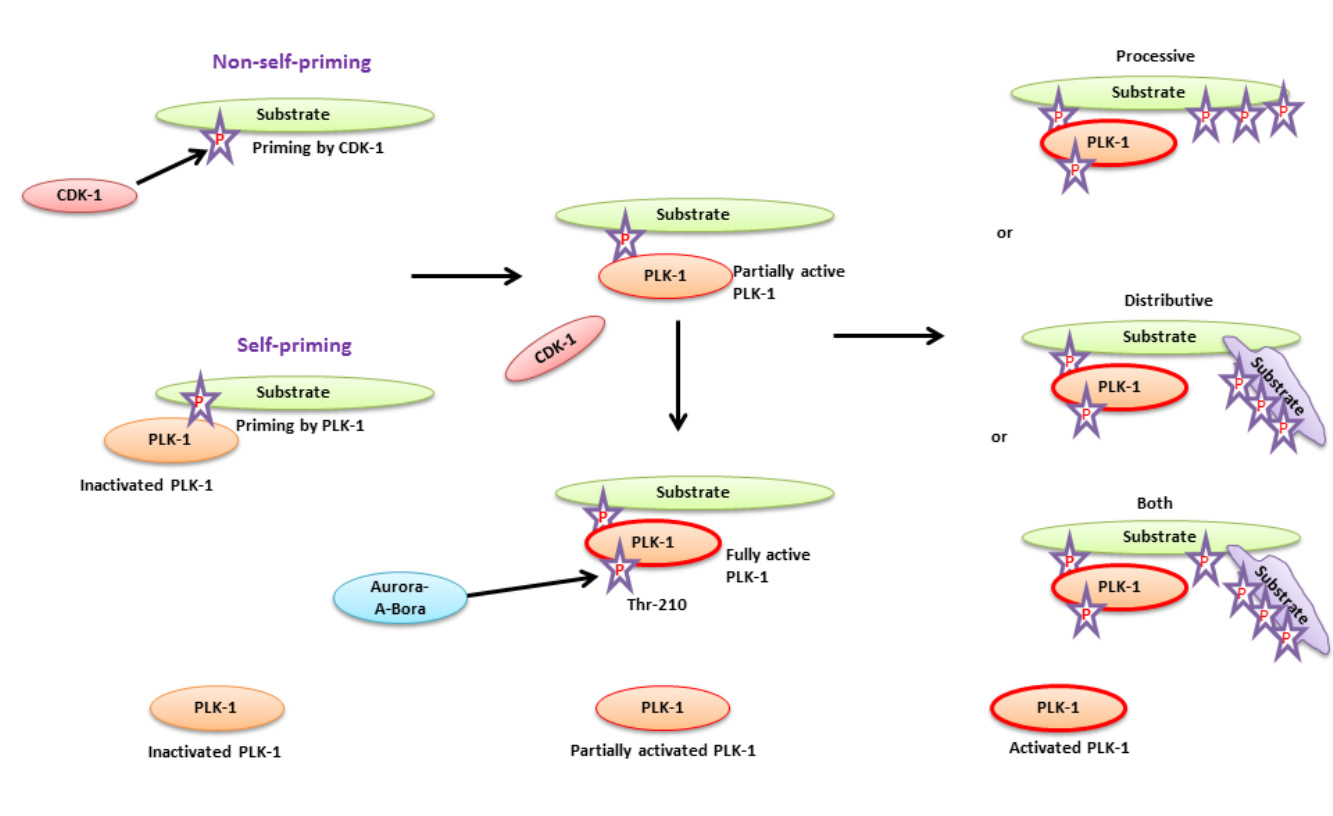
A substrate priming model for PLK-1-targeted substrates during cell cycle - The substrate priming mechanism of PLK-1-targeted substrates mediated either by CDK-1 (non-self-priming) or PLK-1 (self-priming) In non-self-priming, CDK-1 phosphorylates the PLK-1-targeted substrate to facilitate the binding of its PBD to the phosphorylated substrate. PBD-substrate binding is required for partial activation of PLK-1 through physical dissociation of the KD from the PBD. In self-priming, inactivated PLK-1 interacts with a phosphorylated substrate and facilitates its kinase domain dissociation from the PBD. PLK-1 activation is achieved by Aurora-A-Bora to catalyze the phosphorylation substrates. Activated PLK-1 catalyzes phosphorylation of either the same substrate bound to the PBD (processive phosphorylation) or another substrate (binds to the PBD bound substrate, distributive phosphorylation). PLK-1 may also facilitate both models for phosphorylating the targeted substrate at same time.

Furthermore, it has been observed that aberrant expression of PLK-1 also adversely regulates the activity of tumor suppressor genes, *p53* and *pRB,* leading to an override of the checkpoint mechanism [3]. Additionally, suppressed expression of p53 has been described in more than 50% of tumors [3, 13, 14]. Various studies have demonstrated that antisense oligonucleotides, small interfering (si)RNA, phosphopeptides and small molecules-based inhibition of PLK-1 is a promising approach for combating deadly cancer [15–17]. Recently, a study demonstrated that siRNA-based inhibition of PLK-1 causes a notable reduction in the proliferation of cancer cells but not in the proliferation of normal cells [18]. It has been shown that PLK-1 is one of the major players in mitosis during cell cycle progression, and its’ inhibition is a promising approach for combating deadly cancers. Simultaneously, the functional role of PLK-1 is not restricted to only to mitosis regulation because it also has a functional niche beyond mitosis [19]. Outside of its’ traditional function, PLK-1 regulates various events, including DNA replication, transcription, translation, chromosomes dynamics, DNA damage response (DDR), checkpoint adaptation and recovery, neurodegenerative diseases, apoptosis, organogenesis, so on. Studies have confirmed that PLK-1 actively participates either directly or indirectly in DNA replication, transcription and translation [13, 20–27]. During DDR, cells undergo one of three processes. Specifically (1) checkpoint recovery, when cells are allowed adequate time to repair DNA damage and resume the cell cycle for further division; (2) Apoptosis, if DNA damage is irreparable, a checkpoint mechanism activates the ataxia-telangiectasia and rad3-related-cell cycle checkpoint kinase 1/ ataxia-telangiectasia mutated-cell cycle checkpoint kinase 2 (ATR-Chk1/ATM-Chk2) pathways leading to apoptosis in a p53-dependent manner; (3) checkpoint adaptation, whereby damaged DNA is in an irreparable condition and the cell overrides the checkpoint mechanism due to failure of the apoptotic pathways and resumes cell division with damaged DNA, resulting in tumorigenesis [28]. PLK-1 actively participates in the above-mentioned processes [29]. During the S-G2 phase transition, Aurora-A-BORA (Aurora borealis, a co-factor of Aurora kinase A) catalyzes the activation of primed PLK-1 (PLK-1 primed by CDK-1, a cyclin-dependent kinase-1). Aurora-A activates PLK-1 by phosphorylating KD at Thr-210 and enhances the catalytic activity of PLK-1 [30]. Phosphorylated KD of PLK-1 further phosphorylates and regulates the function of targeted proteins, while the PBD of PLK-1 participates in the subcellular localization of activated PLK-1 for facilitating mitosis during the cell cycle [31–33]. PLK-1 also targets a wide variety of substrates that regulate cell cycle progression, and this is achieved either in a CDK-1-dependent (substrates primed by CDK-1) or self-dependent manner (substrates primed by PLK-1) (Figure 1, Table 1). Both the CDK-1-dependent and self-dependent priming of PLK-1 substrates are extensively reviewed in the reference [34].

**Table 1:** The substrates of PLK-1

PLK-1 substrates	PLK-1 phosphorylation site (s)	PLK-1 binding	PBD binding	Determined PBD-binding site (s)	Cdk priming	References
Axin2	DD^S311^MS	+	-	-	-	[58]
Bcl-xl	GYS^23^WSQ, several others	-	-	-	-	[126]
β-Catenin	YRS^718^FHS	+	-	-	-	[127]
BORA	DMS^497^GYN, YNT^501^QNC	+	+*	-	-	[32]
Brca2	DMS^193^WSS, DES^239^LKK	-	-	-	-	[128]
B23	EDS^4^MDM	+	-	-	-	[129]
Bub1	ND	+	+	ST^609^P	+	[130]
BubR1	EDS^676^REA, ELT^792^VIK, EAT^1008^VSV	+	+	ST^620^P	+	[10, 131]
Cdc25C	EFS^198^LKD	+	+	ST^130^P	+	[6, 132]
Cep55	NES^436^LV	+	-	-	-	[133]
CEP170	EDS^676^REA	+	-	-	-	[134]
Claspin	SSS^934^FLT	-	+	ST^906^Q	+	[7]
CLIP-170	SES^195^IS	+	-	-	-	[8]
Cyclin B	ETS^133^GCA, AFS^147^DVI	+	-	-	-	[135, 136]
Emi1	EDS^145^GYS, YSS^149^FSL	-	-	-	-	[137]
FoxM1	NDS^715^LSK, DIS^724^FPG	+	+	ST^596^P, ST^678^P	+	[37]
Grasp65	-	+	+	SS^217^P, several others	+	[138]
GTSE1	RDS^435^CLN	-	-	-	-	[73]
HBO1	DSS^57^PVR	+	+	PT^85^P, VT^88^P	+	[139]
HsCYK-4	DES^149^GSI, DIS^157^FDK, DES^164^LDW, DSS^170^LVK, NES^214^IVA, DST^260^LNS	+	+	SS^170^L, ST^260^L, several others	-	[140, 141]
HSF1	NDS^216^GSA, LFS^419^PSV	+	-	-	-	[142, 143]
IKKb	DQS^733^FTA, DWS^740^WLQ, EHS^750^CL	-	-	-	-	[144]
IRS2 human	RRVS^560^GD, RLS^1109^LM	+	-	-	-	[145]
Kif2A	-	+	-	-	-	[146]
Kif2B	NQT^125^ASG, KIS^204^VLE	-	-	-	-	[146, 147]
Kiz	DLT^379^ISI	+	+	-	-	[148]
MKLP1	RRS^904^STV, RSS^905^TVA	+	+**	-	-	[149]
MKLP2	EHS^528^LQV	+	+	HS^528^L	-	[150]
Myt1	DSS^426^LSS, DDS^435^LGP, DLS^469^DIN, EDT^495^LDP	-	-	-	-	[151]
MyoGF	EDT^574^DED	+	+**	-	-	[152]
Nedd1	TDT^382^LSK, FSS^397^FDD, DES^426^IGK, RYS^637^VNE	+	+	ST^550^P	+	[153]
Nlp	EDS^87^S^88^SLE, ST^161^KEA, EKS^686^QEV	+	-	-	-	[154]
NudC	ENS^274^KLS, DFS^326^KAK	+	+**	-	-	[155]
Optn	EDS^177^FVE	-	-	-	-	[156]
Orc2	SNS^188^ED	+	-	-	-	[92]
p150^Glued^	ELS^179^S	+	-	-	-	[44]
PBIP1	EDS^177^FVE	+	+	ST^78^A	-	[157]
PICH	MTS^1063^KPS	+	+	ST^1063^P	+	[158]
PIN1	KHS^65^QSV	+	-	-	-	[159]
PRC1	AST^578^YSE, HST^602^NIQ	+	+	ST^602^N	-	[160]
PTEN	TDS^385^DP	+	-	-	-	[161]
Ran	AKS^135^IVF	+	-	-	-	[162]
Rictor	ETS^1162^FM	+	-	-	-	[163]
Rock2	DAT^967^IAS, EES^1099^QIR, DSS^1133^SIG, NQS^1374^IRR	+	+	-	-	[164]
Sgt1	NKS^331^FM	+	-	-	-	[165]
TAp73	DST^27^YFD	+	-	-	-	[116]
TCTP	DDS^46^LIG, TES^64^TVI	+	+**	-	-	[26]
Topors	YES^718^SYR	+	-	-	-	[73]
TopoIIa	DFS^1337^DFD, EES^1524^DED	+	-	-	-	[68]
TRF1	ISS^435^DSE	+	+	GT^344^P, VT^371^P	+	[70]
UAP56	-	+	-	-	-	[166, 167]
Vimentn	QDS^82^VDF	+	+	SS^55^P	+	[168]
Wee1A	EDS^53^AFQ	+	+	SS^123^P	+	[102]

+= Phospho-dependent PBD binding, +*=phospho-independent PBD binding, +**= phosphorylation-dependency remains unknown, - not determined

The above-mentioned studies collectively suggest the unconventional role of PLK-1 in replication, transcription, translation, and in DDR, Which are outside of its’ traditional role in mitosis. Moreover, PLK-1 overrides the checkpoint mechanism and causes failure of the ATR/ATM pathways, which leads to tumorigenesis. Therefore, in the present review, we have discuss the role of PLK-1 and the substrates involved in the regulation and control of DNA replication, transcription, translation, ciliogenesis, Alzheimer's disease (AD), checkpoint adaptation, recovery, and apoptosis during DDR.

## PLK-1: REPLICATION, TRANSCRIPTION, AND TRANSLATION

A prerequisite for cell cycle progression is the duplication of the genome, which occurs during the synthesis phase (S- phase) of the cell cycle. A well-controlled replication process is desirable for the faithful duplication of cellular DNA and is regulated by various types of proteins such as the minichromosome maintenance complex (MCM), histone acetyletransferease binding to ORC1 (HBO1), DNA polymerase, PLK-1, among others. The role of PLK-1 during replication has been extensively reviewed by Kumar S. *et al. (2016)* [13]. In brief, PLK-1 catalyzes the initiation and termination of DNA replication during cell cycle progression. In G1 phase, PLK-1 catalyzes the phosphorylation of HBO1 at Ser-57 and facilitates the recruitment of MCM to the Pre-replicative complex (Pre-RC) to generate the Pre-initiation complex. The formation of the Pre-initiation complex is a hallmark for the beginning of DNA replication. In the S-phase, it has been demonstrated that Cdc5 (human PLK-1 orthologue in *Schizosaccharomyces pombe*) phosphorylates ORC2 instead of CDK to initiate DNA replication. Moreover, in G2/M phase, overexpressed Cdc5 mediates hyper-phosphorylation of ORC2, which also terminates DNA replication and allows the cell to enter the transcription phase. These studies collectively provide evidence that PLK-1 acts as a key player during initiation and termination of DNA replication.

Similar to replication, the process of transcription is also a major event that is precisely controlled by several types of transcriptional factors and extracellular proteins. The transcriptional factors, including Forkhead box M1 (FOXM1), are crucial for PLK-1-mediated mitosis regulation. The FOXM1 transcription factor is a widely known mitotic transcription factor that regulates the expression level of G2/M-specific genes, such as cyclin B, Nek2, and PLK-1, and plays a central role in mitosis progression [35]. FOXM1 is a substrate of PLK-1. CDK-1-mediated double phosphorylation of FOXM1 at its’ C-terminus is required for the interaction between PLK-1 and FOXM1. This interaction allows PLK-1 to further phosphorylate FOXM1 and increases FOXM1 activity during G2/M transition. Additionally, the FOXM1-PLK-1 complex also controls the expression of several mitotic-related genes, including PLK-1 itself. Thus, PLK-1-dependent regulation of FOXM1 is not only required for fine tuning PLK-1 regulation but also for generating a positive feedback loop for the coordinated regulation of mitosis [20, 36, 37]. Moreover, a recent article demonstrated that PLK-1 can phosphorylate FOXM1 and causes inhibition of its sumoylation. This event promotes transcriptional activity of FOXM1 to facilitate mitotic progression. This study also mentioned that overexpression of PLK-1 may hyper-regulate the transcriptional activity of FOXM1 to activate the expression of FOXM1 targeted genes during the G2/M phase. Consequently, the misexpression of G2/M-specific genes can cause aneuploidy [27]. Recently, it was observed that PLK-1 can regulate the transcriptional level of tRNA and 5S rRNA genes in a RNA pol-III-dependent manner. These RNAs are extensively required during translation [22]. PLK-1 directly binds to and phosphorylates transcriptional factor protein-1 (Brf-1) on Ser-450 to trigger the expression of tRNA and 5S rRNA during interphase. In addition, PLK-1 also phosphorylates Brf-1 at another site (Thr-270) during mitosis. However, the functional characterization of phosphorylated Brf-1 (Thr-270) by PLK-1 is still unknown. However, PLK-1-mediated phosphorylation of Brf-1 is known to prevent the recruitment of RNA pol-III during mitosis. This means that although PLK-1 can enhance net tRNA and 5S rRNA production in cells through its’ proliferation-stimulating function, the KD of PLK-1 can also reduce the chances of untimely transcription initiation during mitosis *via* the PLK-1-Brf-1 complex. Furthermore, it has also been documented that a PLK-1-mediated phosphorylation site mutant of Brf-1 (mutant Brf-1, Thr-270Ala) causes genomic instability in cells due to inappropriate RNA pol-III activity, which leads to aneuploidy [22].

During the translation process, the mammalian RNA processing protein DExH/D RNA helicase (also known as UAP56) acts both as an mRNA splicing factor and an mRNA export factor. UAP56 is essentially required for mRNA metabolism and axis specification [24, 38]. Previously, it has been demonstrated that the expression levels of PLK-1 and UAP56 are inversely correlated with each other during cell division progression [39]. PLK-1-dependent phosphorylation of UAP56 causes its’ proteasomal degradation and results in mitotic delay as well as sister chromatid cohesin defects, leading to aneuploidy both *in vitro* and *in vivo* [25]. Collectively, these studies concluded that the overexpression of PLK-1 might reduce the level of UAP56 and interfere with mRNA splicing. Furthermore, PLK-1 was also reported to phosphorylate eukaryotic translation initiation factor 4B (eIF4B) in response to arsenic tetroxide treatment in HeLa cells. eIF4B is required for the formation of the pre-initiation complex to initiate translation [21]. Although the study showed that PLK-1 phosphorylates eIF4B, no further detailed mechanism was delineated [21]. It will require additional biochemical studies to clarify the role of PLK-1 in the regulation of translation. In brief, PLK-1 is essentially required for regulating the initiation and termination process of replication by modulating the activity of transcriptional and translational machineries. The dysregulation of PLK-1 may affect the regulatory functions of PLK-1-interacting proteins and their localization during replication, transcription, and translation.

## PLK-1 IN NEURODEGENERATIVE ALZHEIMER'S DISEASE (AD)

**Figure 2:**
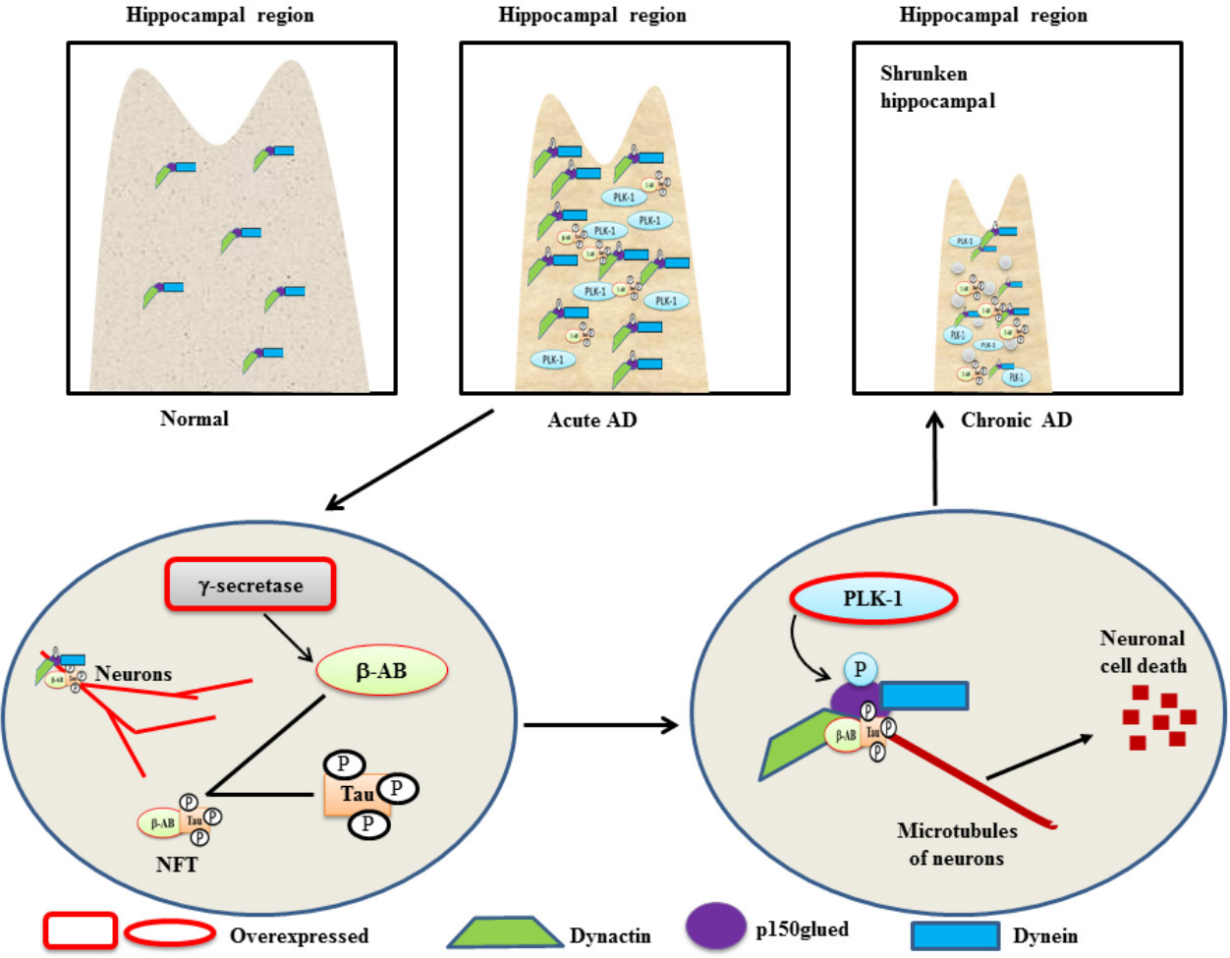
A schematic role of PLK-1-mediated phosphorylation of p150glued in neuronal cell death in a β-amyloid-dependent manner in AD - Overexpressed γ-secretase cleaves mutated β-amyloid to generate a binary complex (NFT) with a hyper-phosphorylated Tau protein The NFT binds to the microtubules of cortical neurons in a phosphorylated p150^glued^ and dynactin-dynein-dependent manner. In the hippocampus of AD patients, overexpression of PLK-1 phosphorylates p150^glued^ in the dynactin and dynein complex to induce neuronal cell death in a NFT-dependent manner. Subsequently, the hippocampal region is shrunken in AD patients because of neuronal cell death.

AD is a progressive neurodegenerative brain disease that is characterized by neuronal cell loss, neuronal inflammation, and a decline in memory and recognition [40]. A mutation in *amyloid precursor protein* (*APP*) can be held responsible for the cleavage of APP through a series of secretase enzymes. Overexpressed γ-secretase activity generates an isoform of a 42aa-long β-amyloid peptide that is deposited outside of neuron bodies and neurofibrillary tangles (NFT) [41]. NFT is an aggregate of hyper-phosphorylated tau proteins and β-amyloids that then binds to microtubules of neurons, resulting in neuronal cell death (Figure 2) [40]. Two major proteins, dynactin and dynein, play indispensable roles in the microtubule dynamics of neurons and in synapse formation during brain development. p150^glued^ is an intermediate component of dynactin and dynein and facilitates the movement of microtubules toward their respective polls during chromosome segregation [42, 43]. PLK-1 has been shown to be localized in susceptible hippocampal and cortical neurons of AD patients [40] and p150^glued^ has been reported to be a substrate of PLK-1 in AD. A study demonstrated that in hippocampal tissues of AD patients, overexpressed PLK-1 phosphorylates p150^glued^ at Ser-179 and induces β-amyloids-mediated neuronal cell death [44]. Moreover, RNAi-mediated knockdown of PLK-1 reduced the β-amyloids deposition in neural cells and inhibited the β-amyloids-mediated neural cell death [45]. This study, for the first time, demonstrated that PLK-1 may be a crucial target for AD therapy, and further studies are required to explore its’ role in AD.

## THE INVOLVEMENT OF PLK-1 IN THE CILIATION CYCLE DURING VERTEBRATE ORGANOGENESIS

**Figure 3:**
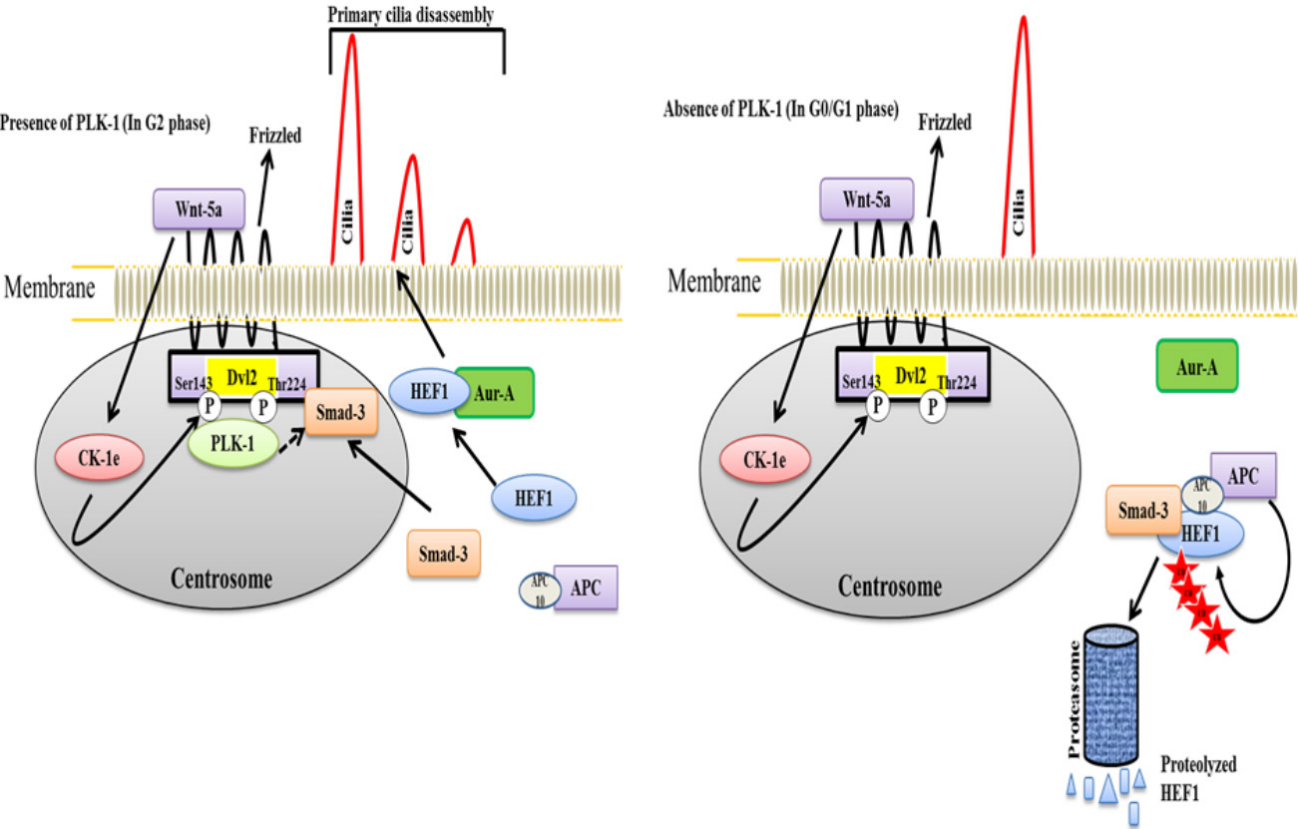
A model illustrating the dynamic cycle of primary cilia disassembly in the presence and absence of PLK-1 a. In the presence of PLK-1, non-canonical Wnt5a triggers the CK-1ε-mediated phosphorylation of Dvl2 to recruit PLK-1, which generates the CK-1ε-PLK-1 complex This complex interacts with Smad-3 and stabilizes the HEF1 for interacting with Aur-A. Because of the unavailability of Smad-3 for generating a HEF1 destruction complex (HEF1-Smad-3-APC complex), stabilized HEF1 interacts with Aur-A to achieve the disassembly of the primary cilia just prior to mitosis. **b**. In the absence of PLK-1, Smad-3 is available for generating the HEF1 destruction complex, HEF1-Smad-3-APC. HEF1 is ubiquitinated and degraded by the proteasome in APC-dependent manner. Consequently, HEF1 is not available to interact with Aur-A to facilitate the disassembly of the primary cilia in the G0/G1 phase.

Proper embryonic development of any animal is a crucial process that is facilitated by various signaling mechanisms. The improper development of an animal causes a wide range of pathological and developmental disorders. During development, cilia are vital structures of stromal and epithelial cells and are involved in the transduction of microtubule-based extracellular signaling. The assembly and disassembly of primary cilia follows a controlled dynamic process throughout the cell cycle. The assembly of cilia is achieved during the G1 phase, while disassembly occurs prior to mitosis [46–49]. The improper function of cilia causes various diseases, including renal cysts, hypertension, diabetes, neuronal, visual, respiratory, and other developmental disorders [50–54]. PLK-1 mediates the disassembly of primary cilia in a non-canonical Wnt5a-Casien kinase1ε(CK1ε)-(Dishevelled2)Dvl2-dependent manner through the activation of the Human Enhancer of Filamentation 1-Aurora-A (HEF-Aur-A) complex [55]. During cilia disassembly, Wnt5a-CK1ε phosphorylates Dvl2 at Ser-143 and Thr-224. Phosphorylated Dvl2 is necessary for the recruitment of PLK-1 and generates the binary complex Dvl2-PLK-1. Formation of the Dvl2-PLK-1 complex enhances Smad-3 binding to Dvl2. The Dvl2-PLK-1-Smad-3 complex stabilizes and augments the level of HEF1 by inhibiting the interaction between HEF1 and Smad-3/APC [56, 57]. This process is required for the establishment of the HEF1 and Aur-A interaction and activates the HEF1-Aur-A complex to facilitate disassembly (Figure 3). Moreover, a mutant of CK-1ε-mediated phosphorylation of Dvl2 demonstrated an increase in the Smad-3-HEF1 interaction by blocking PLK-1 recruitment to Dvl2. This leads to the formation of the Smad-3-HEF1-anaphase promoting complex (APC), which causes the degradation of HEF-1. This study suggested that the Aur-A-HEF1 complex might participate in the activation of PLK-1 and generate a positive feedback loop to regulate the assembly and disassembly of cilia during the cell cycle [55]. Despite the role of PLK-1 in ciliogenesis, a recent study demonstrated that PLK-1 can act as a negative regulator of Wnt/β-catenin signaling [58]. In prostate cancer, siRNA-mediated depletion of PLK-1 increases the cytoplasmic and nucleo-plasmic level of β-catenin in an Axin2-dependent manner. PLK-1 phosphorylates Axin2 at Ser-311 and enhances the interaction of GSK3β and β-catenin, leading to cytosolic degradation of β-catenin in an ubiquitin E3 ligase APC-dependent manner [58].

## THE ROLE OF THE PLK-1 IN CHROMOSOME DYNAMICS

Chromosome dynamics refers to the duplication and faithful segregation of the genome during cell division. Chromosome dynamics is a vital process that is required for the equal and orderly distribution of duplicated sister chromatids into two dividing daughter cells. During mitosis, the condensation of chromatids is a highly regulated event that leads to the segregation of sister chromatids towards their respective poles [19]. The duplication of the genome occurs in S-phase, while the segregation of duplicated sister chromatids is achieved in anaphase. Both the duplication and segregation of sister chromatids are critical and tightly regulated processes to ensure genetic integrity and the equal distribution of replicated genomes into daughter cells [59].

In anaphase, Cdc5 directly phosphorylates and activates 3 regulatory subunits of condensin (an evolutionarily conserved multi-subunit of ATPase), namely, Brn1, Ycg1, and Ycs4. The phosphorylated condensin subunits then initiate the supercoiling activity of DNA. Moreover, phosphorylation mutants of condensin have been shown to cause aberrant chromosome condensation in anaphase and are incompatible with cell survival [60, 61]. In mammals, PLK-1 binds with the PLK-1-interacting checkpoint “helicase” (PICH) and targets PLK-1 on the chromosome arm to maintain the architecture of the chromosome arm during prometaphase. It has been demonstrated that siRNA-mediated PICH depletion leads to the premature release of cohesin proteins (sister-chromatids holding protein in prophase and metaphase) due to the loss of PLK-1 on the chromosome arm, which results in aberrant chromatid segregation [62]. This aberrant chromatid segregation can be prevented by utilizing a Topoisomerase IIα (Topo IIα) inhibitor. This study proposed that PICH-PLK-1 maintains and controls chromatid segregation in a Topo IIα-dependent manner and might play an essential role in genome duplication and segregation during chromosome dynamics.

### Topoisomerase IIα (Topo IIα)

Topo IIα is a DNA gyrase enzyme that plays an essential role in DNA replication, transcription chromosome condensation, and chromatid separation through its’ decatenation activity (decatenation, separating the linkage of two circular DNA strands like a chain before the initiation of replication) [63–65]. Decatenation activity is catalyzed by the Brg/Brahma-associated factors (BAF) complex subunits BRG1 and BAF250A during mitosis. A mutation in the ATPase BRG1 protein has been reported in childhood neuroblastoma and Burkitt lymphomas [66]. Recently, a study reported that the BAF complex can regulate the decatenation activity of newly replicated sister chromatids for ordered segregation during mitosis [67]. Moreover, the expression of point mutated BRG1 has been reported in human tumors. Specifically, a mutated BRG1 has been found to form an anaphase bridge (sister chromatids attached by catenated DNA strands), which subsequently leads to a block at the G2/M transition (represents decatenation of checkpoint). Meanwhile, BAF250A catalyzes the interaction of the BAF complex to Topo IIα to recruit Topo IIα to DNA [67]. This study observed that Topo IIα and DNA binding is facilitated by the ATPase activity of BRG1. The binding between Topo IIα and DNA prevents DNA entanglement during mitosis through BAF complexes, suggesting that BAF acts as a tumor suppressor protein. Another study has demonstrated that PLK-1 phosphorylates Topo IIα at Ser-1337 and Ser-1524 to control the segregation of sister chromatids by increasing its’ catenation activity [68]. Overexpression of mutant Topo IIα which lacks phosphorylation site for PLK-1, causes S-phase arrest, which suggests that PLK-1-mediated phosphorylation of Topo IIα is primarily achieved during S-phase. Meanwhile, mutant Topo IIα can also activate ATM/ATR-dependent DNA damage checkpoint kinases. This activation might be possible because Topo IIα activity is inhibited. This study also demonstrated that PLK-1-mediated phosphorylation of Topo IIα rescued the defects induced by the depletion of Topo IIα during sister chromatid segregation. This study also showed that phosphorylation by PLK-1 is essential for catalyzing the decatenation activity of Topo IIα during mitosis. Collectively, these studies provide evidence that PLK-1-associated Topo IIα phosphorylation may regulate sister chromatid segregation in a BRF and BRG1-dependent manner.

### Telomeric-repeat binding factor (Trf-1)

The Trf1 protein is a component of the telomere nucleoprotein complex that regulates cell cycle progression and inhibits telomerase activity. During cell division, Trf1 maintains the length of the telemetric end of the chromosomes [69]. Recently, it was identified that Trf1 acts as a substrate of PLK-1. It was observed that PLK-1 physically interacts with Trf1 to phosphorylate it at Ser-435 after phosphorylation by CDK-1 *in vivo* [70]. In validation, it has been shown that Trf1-Ser-435A mutant does not induce apoptosis in short telomere ends containing cells as well as not in long telomere end containing cells. In contrast, diminishing levels of Trf1 still induces apoptosis in short telomere end containing cells but not in long telomere end containing cells [70]. Surprisingly, RNAi-mediated knockdown of Trf1 induced apoptosis in cells that even had short telomere ends. PLK-1-mediated phosphorylation of Trf1 can increase the DNA binding ability of Trf1, and this binding ability reaches its’ peak both *in-vivo* and *in-vitro* during mitosis. Moreover, this study recommended that PLK-1-dependent phosphorylation of Trf1 is critical not only for cell cycle regulation but, also for telomere end length control.

## PLK-1 IN p53 AND pRB REGULATION

Both p53 and pRB are well-known tumor suppressor genes that have been reported to be down-regulated in a wide range of human cancers. Apoptosis is not induced in cancer cells having downregulated expression of p53, and pRB [3]. Overexpression of PLK-1 downregulates p53 expression both directly and indirectly [3, 71]. It has been shown that PLK-1 can physically bind and phosphorylate p53, which inhibits its’ transactivation and pro-apoptotic function by obstructing the expression of p53-targeted, apoptosis-inducing genes, such as p21 and BAX [72]. Studies have also demonstrated that PLK-1 regulates p53 expression in a TOPORS and GTSE1-dependent manner, which results in the inhibition of p53 functions [73–75]. Human papilloma virus type-16's onco-proteins (E6 and E7) possess the ability to transform normal cells into cancer cells [76, 77]. In human keratinocytes, due to failure of spindle checkpoint by HPV-E6/7 leads to polyploidy. Moreover, HPV-E6/7 can inhibit the transcriptional expression of *p53* and *pRB*, respectively [77]. Induction of HPV-E7 aberrantly enhances the expression of mitosis-regulating genes, including PLK-1. Overexpressed PLK-1 causes cancer in epithelial cells of lower genital tract [76]. Whereas, HPV-E6 binds with p53 to make a ternary complex with E6AP (an E3 ubiquitin ligase) leading to p53 degradation and pRB functional impairment [77]. These studies collectively provide evidence that HPV-E6/7 may transform the normal cells into malignant cells by inhibiting p53 and pRB expression in a PLK-1-dependent manner.

### TOPORS (Topoisomerase I Binding, Arginine/Serine-Rich, E3 Ubiquitin Protein Ligase)

TOPORS is identified as a DNA topoisomerase I binding protein and contains both ubiquitin and SUMO-1 E3 ligase activity [78–80]. TOPORS comprises an N-terminal RING Zinc-finger domain that is closely related to the homology of C3HC4-RING domains of viral proteins and is associated with viral transcription regulation [80]. Recently, TOPORS has also been reported to be a p53 binding protein [78]. Expression level of TOPORS is high in human testis, but its’ expression is diminished or undetectable in different types of cancers, including colon, lung, and brain [81]. The overexpression of TOPORS is associated with cancer cell death due to its involvement in G0/G1 arrest and apoptosis induction, which explains why it has diminished or undetectable expression levels in cancer cells [81, 82]. Studies have demonstrated that TOPORS can also sumoylate and degrade transcription factors, including p53, hairy, Topo I, Sin3A, and NKX3.1 [79, 83–85]. Recently, in mouse ovarian granulosa cells, it was observed that TOPORS-mediated sumoylation of p53 (Lys-375) causes its’ translocation into the nucleus and enhances its’ stability. Increased stability of p53 promotes the pro-apoptotic function of p53. However, mutated p53 (loss of sumoylation site, Lys-375R) is localized both in the nucleus and cytoplasm, which suggests that nuclear localization and stability of p53 requires TOPORS-mediated sumoylation [86]. Therefore, TOPORS might be regarded as a tumor suppressor protein because it has dual activity in ubiquitination and sumoylation.

**Figure 4:**
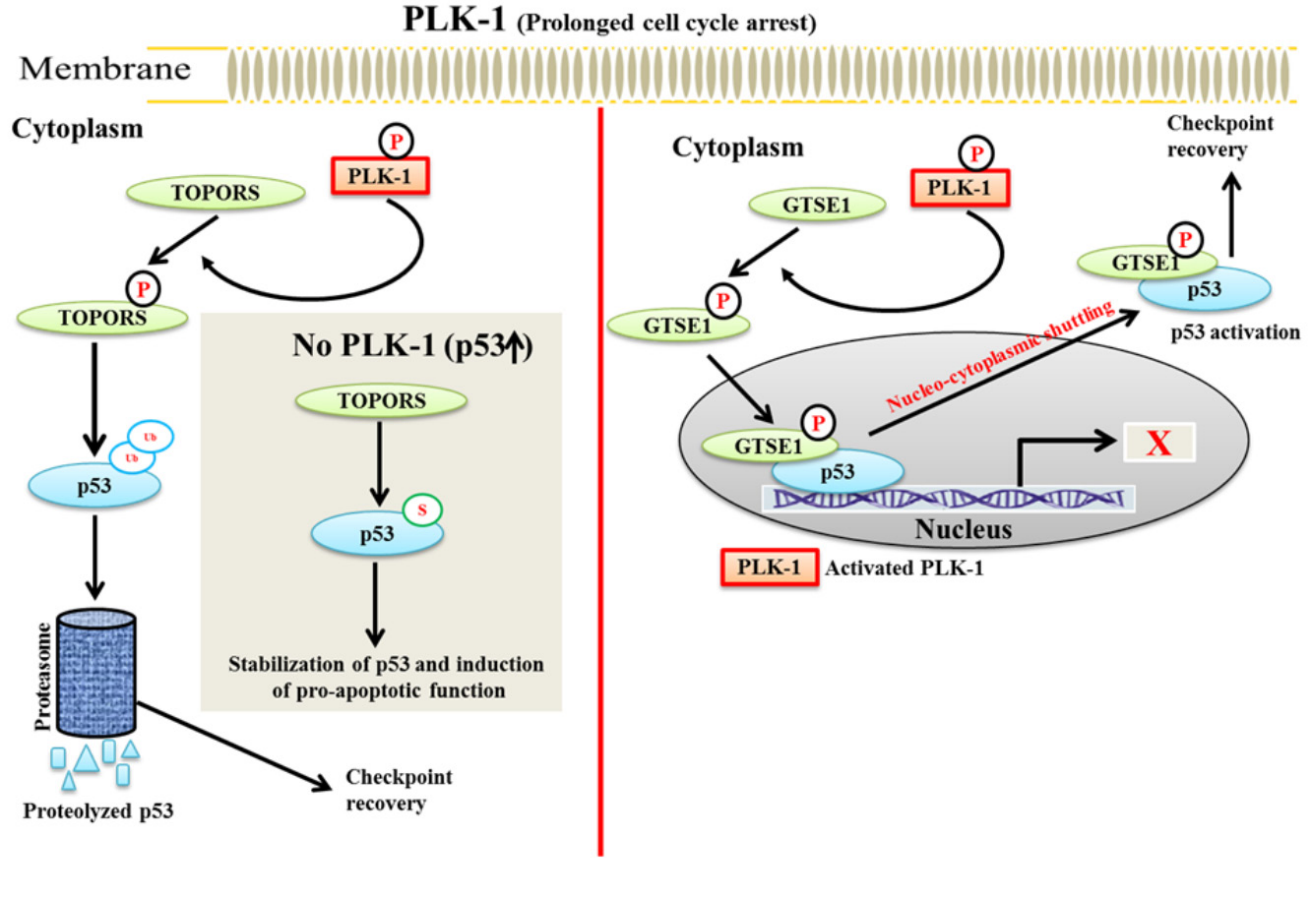
A schematic model of PLK-1-mediated regulation of p53 in TOPORS and GTSE1-dependent manner - During prolonged cell cycle arrest, a. activated PLK-1 phosphorylates TOPORS to facilitate the ubiquitination and proteasomal degradation of p53, which promotes checkpoint recovery During normal cell cycle progression, TOPORS sumoylates and stabilizes p53 for inducing its pro-apoptotic functions. **b**. PLK-1 phosphorylates and translocate GTSE1 into the nucleus. Nucleated GTSE1 binds to the C-terminus of p53 and catalyzes the shuttling of p53 from the nucleus to the cytoplasm. GTSE1-dependent shuttling of p53 results in inactivation of p53, which leads to checkpoint recovery.

It has been observed that TOPORS can be a substrate of the proto-oncogene PLK-1 [75]. Overexpressed PLK-1 phosphorylates TOPORS (Ser-718), inhibits its’ sumoylation activity and enhances its’ ubiquitination activity. Phosphorylated TOPORS then ubiquitinate p53 and causing its’ degradation (Figure 4a). A phosphorylation mutant of TOPORS (Ser-718A) causes the accumulation of p53. These results demonstrated that PLK-1 negatively regulates p53 stability in a TOPORS-dependent manner and degrades p53 by increasing the ubiquitination activity of TOPORS, which leads to tumorigenesis.

### GTSE1 (G2 and S-phase-expressed 1)

GTSE1 was initially isolated by a screening of p53-inducible genes. The expression of PLK-1 is maximum during S and G2 phases of the cell cycle and is normally localized at the microtubule network. During G2 phase, the overexpression of p53 induces endogenous expression of GTSE1 and delays the G2/M transition [87].

GTSE1 is a direct substrate of PLK-1. During DNA damage, PLK-1 phosphorylates GTSE1 (Ser-435) and facilitates its translocation into the nucleus. Phosphorylated GTSE1 binds to the C-terminal domain of p53 and facilitates the nuclear-cytoplasmic shuttling of p53 from the nucleus to the cytoplasm. Consequently, p53 is degraded and leads to a failure of apoptosis [88, 89]. During DNA damage, GTSE1 may have dual role in override of the G2/M checkpoint. Specifically, it may either cause a delay in the G2/M transition or it may lead to a failure of apoptosis induction. These studies imply that PLK-1-phosphorylated TOPORS and GTSE1 can negatively regulate the p53 pathway during the DDR damage response (Figure 4b) [73, 75]. The role of GTSE1 is extensively discussed below in the checkpoint recovery section under the DDR.

## PLK-1: DRUG RESISTANCE

Drug resistance to molecular and chemotherapeutic-targeted therapies is one of the major issues during current era of cancer research and is an obstacle for complete eradication of cancer [90]. Drug resistance may arise either prior to chemotherapy (Primary resistance) or as a result of chemotherapeutic treatment (Secondary or Acquired resistance) [91]. Moreover, a cancer cell can develop drug resistance by different type of molecular alterations, including drug transport and metabolism, drug inactivation, mutation, amplification of drug targets and genetic rewiring (which can cause impaired apoptosis) [90, 91]. Development of cancer is a result of aberrant cell cycle progression, which is generally governed by two major sets of genes, i.e. proto-oncogene and tumor suppressor genes. The genetic alteration or mutation (either upregulation of proto-oncogenes or downregulation of tumor suppressor genes) in these genes can transform normal cells into cancer cells. Studies have demonstrated that PLK-1 is overexpressed in more than 50% type of cancers. siRNA-mediated depletion of PLK-1 inhibits the tumor growth, both *in vivo* and *in vitro* [13, 14]. Furthermore, studies have demonstrated that overexpressed level of PLK-1 is not only associated with tumor progression but it may also lead to drug resistance. In pancreatic cancer, PLK-1 acts as a mediator for inducing gemcitabine resistance (replication poison) [92]. Gemcitabine treatment halts DNA synthesis and arrest cell cycle in G1/S phase [93]. On the other hand, PLK-1 is responsible for proper DNA synthesis by phosphorylating ORC2 (Origin recognition complex2) and HBO1 (histone acetyltransferase binding to ORC1 protein). Hence, elevated levels of PLK-1 can cause gemcitabine resistance in pancreatic cancer cells and xenograft tumor by restoring the blocked DNA synthesis. Furthermore, PLK-1-mediated phosphorylation of Hbo1 increases cFos (a component of AP-1 transcription factor) expression, resulting in elevated expression level of multidrug resistance 1 (MDR1) protein (MDR1 is an ABC type transporter which can efflux the drugs from cell into extracellular space). Hence, depletion of MDR1 or cFos enhances the sensitivity and efficacy of gemcitabine to gemcitabine-resistance pancreatic cancer cells [9, 92]. Another study reported that small molecule (BI2536)-mediated inhibition of PLK-1 potentiates the anticancer activity of metformin (oxidative phosphorylation and mTORC1 inhibitor) in prostate cancer cells [94]. Inhibition of PLK-1 increases the sensitivity of metformin through stabilizing the level of p53, resulting in initiation of p53/Redd1-dependent apoptosis in prostate cancer cells [94]. Metformin has been shown to induce glycolysis in diabetic patients to reduce blood glucose level and enhances insulin sensitivity [95]. BI2536 (PLK-1 inhibitor)-treatment can inhibit the metformin-induced glycolysis and glutamine anaplerosis (glutamine-dependent cancer cell survival). These studies suggested that inhibition of PLK-1 improves metformin anticancer activity. Study also reported the involvement of PLK-1 in transition of androgen-dependent prostate cancer to castration-resistant prostate cancer (CRPC; an androgen-independent prostate cancer) by causing glutamine anaplerosis. Another study documented that inhibition of PLK-1 can sensitize the CRPC to androgen signaling inhibitors and can increase the efficacy of androgen signaling inhibitors by blocking androgen signaling, *in vivo* and *in vitro* [96]. From these evidences it appears that PLK-1 plays a major role in inducing drug resistant in different type of cancers. Hence, strategies of inhibiting PLK-1 may be utilized for sensitizing the resistant cancer cells to targeted drug therapies.

## PLK-1: DNA DAMAGE RESPONSE (DDR)

DNA damage is an aberrant event that breaks the integrity of genomic DNA. Accumulation of DNA damages may transform a normal cell into a cancer cell. In the presence of damaged DNA, when DNA breaks or replication spoilage occurs in the genetic material, the checkpoint machinery is activated to delay cell cycle progression and allow adequate time to repair the damage to the DNA to maintain genomic integrity [97]. The checkpoint machinery is highly conserved throughout eukaryotes, and any mutation and/or genetic defects that occur in the cells may cause them to become highly susceptible to transformation, which can lead to cancer [98]. During normal mitosis progression, CDK-1 and cyclin B play a crucial role during G2/M transition. CDK-1 interacts and binds to Cyclin B to make a binary complex, the CDK-1-Cyclin B complex. The CDK-1-Cyclin B complex is found in an inactivated form during the G2 phase owing to the negative phosphorylation of CDK-1 at Thr-14 and Tyr-15. Inhibitory phosphorylation of CDK-1 is catalyzed by WEE1 and MYT1. WEE1 is a Ser/Thr nuclear kinase that regulates the entry of the cell cycle into mitosis while MYT1 is a cell cycle regulated tyrosine kinase which inhibits the activation of CDK-1. Nevertheless, during the course of G2/M transition, activated PLK-1 also phosphorylate and stimulates the dephosphorylation activity of CDC25A to activate CDK-1-Cyclin B complex to ensure an orderly G2/M transition [3, 99]. Meanwhile, PLK-1 can also phosphorylate WEE1 (Ser-53) and MYT1 (Ser-426) and inhibit their regulatory function by activating the CDK-1-Cyclin B complex. Conversely, when an extended cell cycle arrest occurs during the DDR, overexpressed PLK-1 may activate CDC25A and facilitate the activation of the CDK-1-Cyclin B complex [100–103]. The activated CDK-1-Cyclin B complex overrides the G2/M checkpoint and causes premature mitotic entry, which results in tumorigenesis [28, 40]. In DDR, the overexpression of PLK-1 inhibits the function of p53, but it a well-known protein that maintains genomic integrity and activates the repair machinery in the can activate CDK-1 and CDC25A to promote the G2/M transition. As a consequence, the cell fails to undergo apoptosis or delay cell cycle progression to repair the damaged DNA [11, 28, 104].

**Figure 5:**
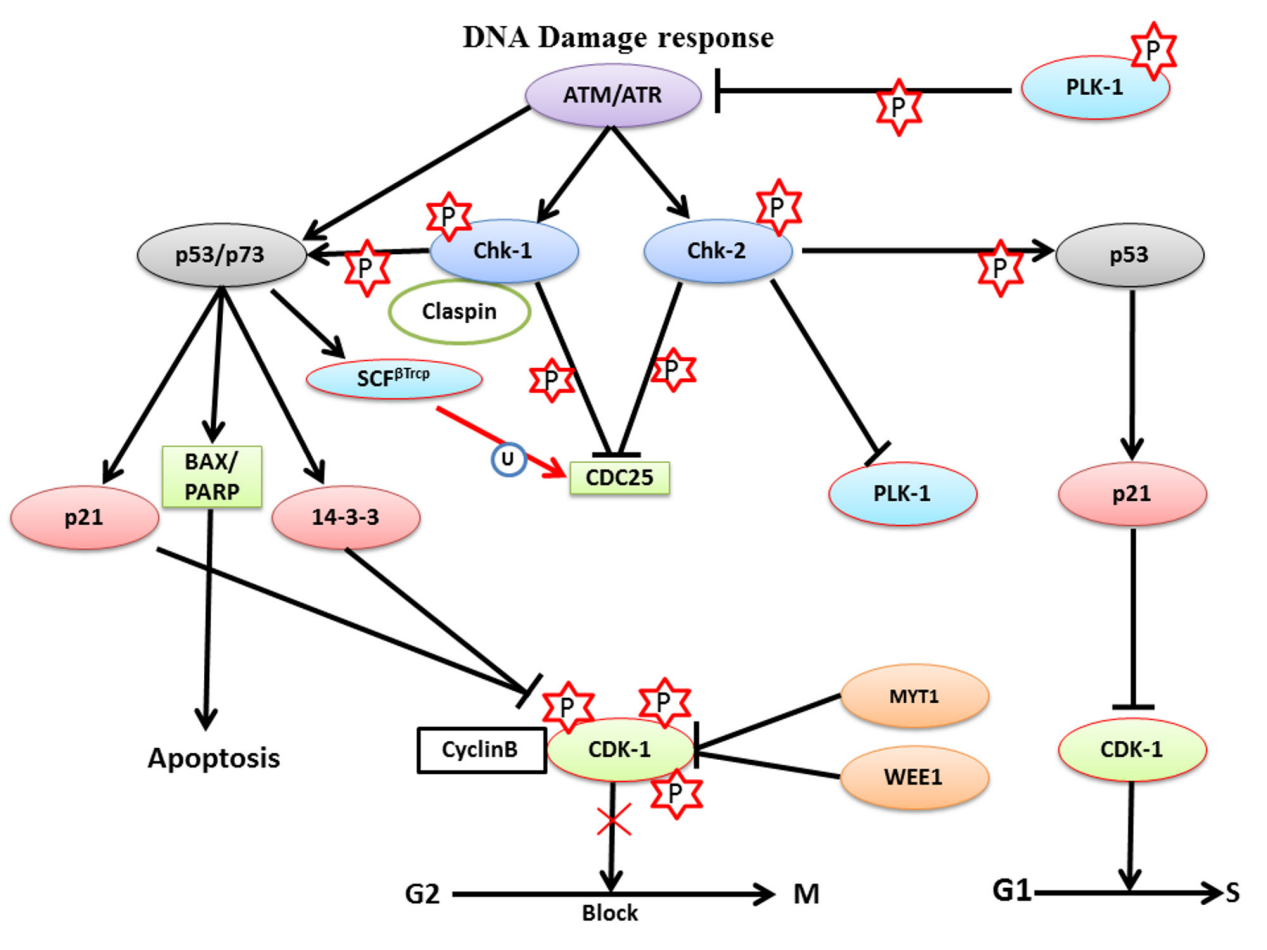
The role of PLK-1 in the DNA damage response- During the DNA damage response, ATM or ATR kinases delay cell cycle progression to provide adequate time to repair the damage in a Chk-1- or Chk-2-dependent manner, respectively The ATR-Chk-2 complex inhibits PLK-1 and CDC25 to block the G2/M transition. Moreover, ATR-Chk-2 catalyzes p53 phosphorylation to inhibit CDK-1, which facilitates the G1/S transition. Simultaneously, ATM-Chk-1 triggers p53 to activate its’ downstream targets p21 and 14-3-3. p21 and 14-3-3 both inhibit the CDK-1-cyclin B1 binary complex, which is required for the G2/M transition and, arrests cells in the G2/M phase for short periods of time. Inhibition of the G2/M transition provides time for damage repair and recovery from DNA damage.

DNA damage is mainly of two types: single-strand breaks (SSBs) and double-strand breaks (DSBs). Among these, DSBs are more dangerous to the integrity and survival of the cells. During DDR, the checkpoint machinery activates two main pathways, ATR-Chk-1 pathway and the ATM-Chk-2, to repair the different types of DNA damages. The ATR-Chk-1 pathway is involved in ultraviolet (UV)-radiation-mediated DNA damage repair while both, ATR-Chk-1 and ATM-Chk-2 pathways, are required to repair DSBs and delay replication forks. During DDR, ATM recruits Chk-2 at the damaged site and phosphorylates it so that it can activate p53. Meanwhile ATR phosphorylates Chk-1 to interact with claspin so that it can activate p53. Activated p53 can in turn activate p21 (endogenous inhibitor of CDK-1 inhibitor) to inhibit CDK-1 during the G1/S transition, which delays cell cycle progression. During the G2/M transition, ATM-Chk-2 phosphorylates and inhibits CDC25A (Ser-123) to block the G2/M transition because CDK-1 is not activated. Activated p53 can also lead to degradation of CDC25A in a Skp1-Cul1-F-box ubiquitin ligase (SCF^βTrcp^)-dependent manner to inhibit the G2/M transition (Figure 5). These studies collectively suggested that both, ATR-Chk-1 and ATM-Chk-2, the pathways could block the catalytic activity of CDC25A and CDK-1 to delay mitosis progression through p53 [105–108].

When cells are arrest in the G2 phase during DDR, the cells can follow one of three possible routes. First is checkpoint recovery, in which the cells allow additional time for activating DNA damage repair pathways and repairs the damaged DNA before commencement of segregation process. The second option is apoptosis, in which the cells activate apoptosis-inducing pathways to induce cell death, if DNA damage is irreparable. The third choice is a potentially deadly decision known as checkpoint adaptation, in which stressed cells progresses to divide with damaged DNA and may result in malignant transformation. Surprisingly, PLK-1 has been found to be associated with all three processes [109–111].

### PLK-1: checkpoint recovery

Multicellular organisms prefer a better and less mutagenic strategy to avoid the harmful consequences of DNA damage. Mammalian cells can delay cell cycle progression to allow adequate time for repairing the damaged DNA prior to resuming cell division. This phenomenon is commonly known as checkpoint recovery. If DNA damage occurs in the G2 phase, activation of p53 by the ATM-Chk-2/ATR-Chk-1 pathway delays the replication process and provides adequate time for repairing damaged DNA [110]. The events regulated by the above-mentioned pathways for DNA repair along with p53 have been discussed in a previous section. To elucidate the involvement of various proteins involved in the ATM-Chk-2 pathway, a combination of bioinformatics and biochemical approaches are a prerequisite. Recently, a study identified various PLK-1 substrates, including 53BP1, which participates in G2/M DNA damage checkpoint silencing [111]. CDK-1-dependent priming of 53BP1 is required to generate a docking site for PLK-1. PLK-1 phosphorylates 53BP1 at this docking site. During ionization radiation (IR)-mediated cell cycle arrest, the 53BP1 protein is required to release checkpoint arrest. PLK-1 interacts with the 53BP1 protein to inactivate the DNA damage checkpoint and release cells from IR-mediated arrest. Moreover, PLK-1 can also phosphorylate and inactivate the 53BP1-binding protein Chk-2. This was further confirmed when a mutant 53BP1 protein was not able to interact with PLK-1 and prevent the release of cells from cell cycle arrest or restart IR-mediated cell cycle arrest [111]. Taken together, the PLK-1-mediated negative feedback loop is triggered by ATM-Chk-2 regulation through the phosphorylation of 53BP1 and Chk-2. This means that PLK-1 inactivates G2/M checkpoint signaling to control the duration of the checkpoint.

**Figure 6:**
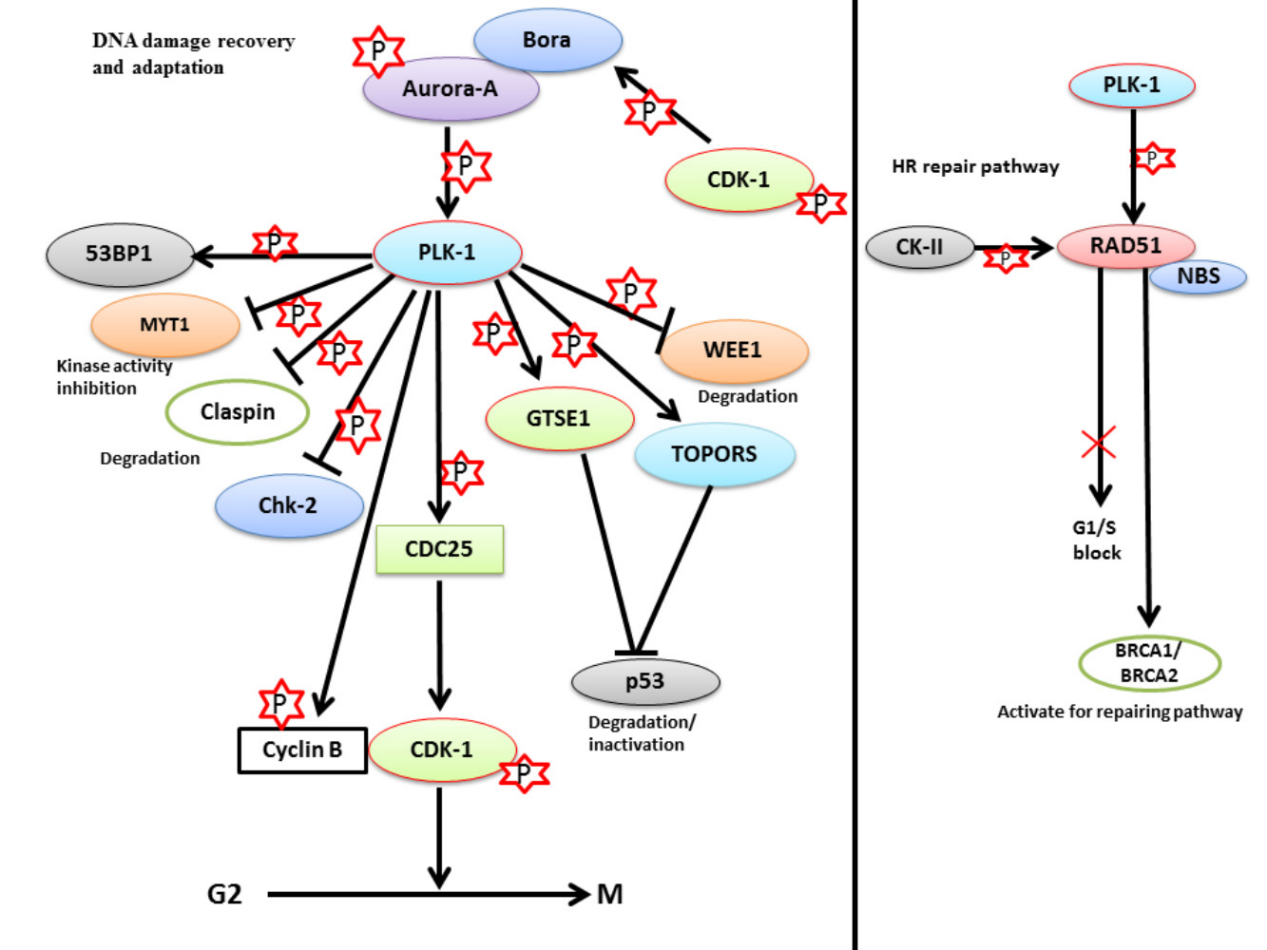
The role of PLK-1 in checkpoint adaptation and recovery - a. During prolonged cell cycle arrest, PLK-1 is upregulated in cells that are stressed for a long period of time Up-regulated PLK-1 inhibits the damage repair mechanism and releases the cells from G2/M arrest with damaged DNA. PLK-1 inhibits/degrades the various proteins that are associated either with the damage repair mechanism or with G2/M arrest, including Chk-2, p53, MYT1, WEE1, and claspin. PLK-1 also phosphorylates CDC25 to activate its’ phosphatase activity. Phosphorylated CDC25 activates the CDK-1-cyclin B1 complex to release the cell cycle from G2/M arrest with damaged DNA. **b**. In the HR repair pathway, PLK-1 phosphorylates RAD51 to activate BRCA1/2 for activating the repair pathways in a CK-II-dependent manner. Phosphorylated RAD51 interacts with NBS, and the RAD51-NBS complex is recruited to the damage site to repair the damage. Moreover, PLK-1 and CK-II-dependent phosphorylation blocks the G1/S transition and provides time for damage repair.

To regulate the checkpoint recovery process, silencing of p53 is a prerequisite. Ideally, p53 should be functionally active to sustain G2/M arrest following DNA damage. A question that arises is how p53 is inactivated during G2/M checkpoint recovery. To answer this question, Liu *et al*. identified a novel substrate of PLK-1, the GTSE1 protein, which negatively regulates the level of p53 during checkpoint recovery. GTSE1 is primarily expressed during the G2 and S-phase and is localized in the cytoplasm. During checkpoint recovery, PLK-1-mediated phosphorylation of GTSE1 (Ser-435) is required for checkpoint recovery and its’ nuclear translocation [19, 73]. In the nucleus, GTSE1 binds to p53 and triggers the shuttling of the GTSE1-p53 complex to the cytoplasm. The shuttling of p53 to the cytoplasm causes its’ degradation or inactivation during DDR. However, the molecular mechanism of GTSE1-dependent degradation of p53 during the process of checkpoint recovery is largely unknown. GTSE1-mediated degradation of p53 leads to the activation of CDK-1-cyclin B complex, which releases the G2/M-arrested cell cycle to promote mitotic entry. In addition, cells with PLK-1 phosphorylation deficient mutant of GTSE1 (Ser-435-GTSE1-Ala) displayed reduced nuclear accumulation of GTSE1, signifying that PLK-1-mediated phosphorylation of GTSE1 may play a significant role in G2/M checkpoint recovery through the elimination of p53 (Figure 6a) [73].

### PLK-1: in apoptosis

Apoptosis is an indispensable suicidal process for organogenesis, cell proliferation, and maintaining tissue homeostasis in all multicellular organisms. Moreover, apoptosis is also required for the removal of damaged and aberrant cells. The ordered regulation of apoptosis is critically required for maintaining the balance between cell survival and cell death to prevent various diseases, such as cancer [112, 113]. p53 is a major key player which is required for apoptosis induction. During DDR, checkpoint machinery activates and phosphorylates p53, which leads to apoptosis. Overexpression of PLK-1 negatively regulates p53 activity in a TOPORS and GTSE-1-dependent manner. Moreover, studies have demonstrated that small molecule, such as BI2536, and phosphopeptides, and siRNA-mediated inhibition of PLK-1 may trigger both p53-dependent and p53-independent apoptotic pathways to inhibit tumor progression [15–17]. It was observed that the antisense phosphorothioate oligodeoxynucleotide for PLK-1 inhibits p53 expression in colon cancer, both at the transcriptional and translational levels, to induce apoptosis in a caspase-dependent manner [114]. Studies have collectively proposed that inhibition of PLK-1 may activate apoptosis through the induction of p53 and caspase cascade activation.

p73 is a member of the p53 transcription family that is required for cell cycle regulation and apoptosis. p73 may positively regulate the apoptotic function of p53-regulated genes. PLK-1 catalyzes the phosphorylation (Thr-27) and inactivation of p73 to inhibit p73-dependent activation of apoptotic proteins, including 14-3-3σ, p21^CIP/WAF1^ and Bax. It was found that siRNA-mediated knockdown of endogenous PLK-1 in *p53*-/- cells causes activation of p73 and PARP to arrest cells in sub-G1 phase [115, 116]. Furthermore, an extended down-regulation of PLK-1 expression enhances the level of p53, Bax, and p21^CIP/WAF1^ and localizes p21^CIP/WAF1^ to the cytoplasm and nucleus, which results in G2/M arrest through inhibition of CDK-1. A reduction in PLK-1 expression may also enhance the sensitivity of cancer cells to anti-cancer agents such as cisplatin [117]. siRNA-mediated knockdown of PLK-1 results in induction of apoptosis through caspase-3 activation and fragmentation of the nuclei in PLK-1-overexpressing cancerous cells.

### PLK-1: checkpoint adaptation

Checkpoint adaptation is a lethal process for the survival of multicellular organism. Multicellular organisms can maintain the integrity of their genome by activating checkpoint machinery to delay phase-transition and allow time to repair damage. In interphase, cells with DNA damage have activated ATR/ATM pathways, which provide adequate time to repair damaged DNA. This process enhances the chance that the cell will survive from the fatal changes that occurred during cell division and relaxes checkpoint stress to move the cells into the next phase of the cell cycle [28, 99]. However, in budding yeast, stressed cells (with damaged DNA) are permitted to override the G2/M checkpoint barrier, which allows stressed cells to enter in cell division without repairing damaged DNA. This phenomenon is widely designated as “adaptation”. The phenomenon of adaptation may be useful for unicellular organisms to enhance their chance of survival in the presence of damaged DNA [118]. The DNA damage-mediated G2/M checkpoint adaptation was first time verified in a haploid strain of *S. cerevisiae*. Haploid yeast have a non-essential extra copy of chromosome VII that contains a cleavage site for the activity of homothallic switching endonuclease (HO) in the *MAT* locus. The product of *Rad52* activates repairing mechanism in response to HO-mediated damage in *MAT locus*, while *Rad52* mutant haploid yeast is unable to activate repair machinery for restoring the HO-induced damages. When *Rad52* mutant haploid yeast is arrested in G2/M phase for approximately 10 h, cells can override the G2/M checkpoint barrier in response to extended arrest and re-enter the cell cycle for further division with HO-mediated DSBs [119]. Moreover, this study observed that Cdc5 (human PLK-1 homolog) regulates the checkpoint adaptation in yeast. Sandell *et al*. (1993) also isolated two adaptation-defective mutants (*Bad1* and *CKB2*) from *Rad52* mutagenized colonies of yeast that were permanently paused during G2/M phase. The first mutant, Bad1 (break adaptation-defective), was allelic to *CDC5* and hence was referred to as *cdc5-ad*. It was permanently halted during G2/M phase in response to irreparable DNA damage. The second mutant, *CKB2*, was also permanently arrested at the G2/M phase but was affected at the *CKB2* locus (encoding a non-essential subunit of casein kinase-II). *cdc5-ad* appears as a large-bud shaped structure with irreparable DSBs on chromosome VII due to a mutation in *Rad52*. However surprisingly, it was observed that the mutant *cdc5-ad*-mediated G2/M arrest was completely released by knockdown of the *Rad-9* gene [119, 120]. Recently, it was demonstrated that overexpression of Cdc5 leads to an override of the G2/M checkpoint barrier, which is triggered by DSBs [121]. Moreover, overexpression of Cdc5 inhibits Mec1/ATR activity and also regulates various proteins downstream of Mec1/ATR such as transducer kinase Rad53/Chk-2, Ddc2/ATRIP, and checkpoint-mediator Rad-9 (Table 2). Additionally, up-regulated expression of Cdc5 slows the DSB repair mechanism by phosphorylating Rad-9, which leads to its’ inhibition. In summary, these findings proposed that Cdc5 may act as a central player to release G2/M checkpoint arrest in the presence of damaged DNA by inhibiting Rad-9 [121].

**Table 2:** PLK-1 regulated mechanisms underlying checkpoint adaptation and checkpoint recovery in human

PLK-1-mediated events	Checkpoint adaptation (Human, Yeast, Xenopus)	Checkpoint recovery (Human, Yeast)
Degradation of Claspin	Yes	Yes
Cdc25 and WEE1 inactivation	Yes	No
Chk-2 and 53BP1 inactivation	Yes	No
Nuclear accumulation of GTSE1	Yes	Yes
Inhibition of Mec1/ATR targets (ATRIP, Rad-9, Rad-53/Chk-2) phosphorylation	No	Yes
Regulation of Sae2/CtIP function	No	Yes
Shuttles of p53 form nucleus to cytoplasm and degrades	No	Yes

Haploid yeast cells demonstrate that unicellular organisms can divide with damaged DNA, which increases its’ chances for survival. However, while checkpoint adaptation is beneficial for single-celled eukaryotes, its functional role in multicellular eukaryotes remains unclear. However, a recent study demonstrated that human osteosarcoma cells can override IR-induced G2/M checkpoint in a Chk-1-dependent manner and can enter mitosis with an increased expression level of γ-H2AX foci (a marker representing irreparable DSBs in cells) [122]. Moreover, it has been observed that the G2/M checkpoint override process is regulated by PLK-1 in the presence of DSBs. The G2/M checkpoint override process may be delayed either by PLK-1 inhibition or by Chk-1 overexpression [122]. Therefore, these findings collectively demonstrate that PLK-1 can directly regulate G2/M checkpoint override in DNA damage-containing human cells and provides evidences of checkpoint arrest override for further cell divisions in multicellular eukaryotes.

Conversely, PLK-1 also plays a pivotal role in DNA damage repair during S and G2 phase through homologous recombination (HR) [123–125]. Nevertheless, the regulatory mechanism of the HR-mediated repair pathway is not fully understood. HR is an active process for repairing damaged DNA during S and G2 phases. In the HR repair pathway, many proteins are involved, including Rad-51 (a highly conserved protein from yeast to humans). Rad-51 is a recombinase which actively participates in the repair of DSBs [124]. PLK-1 phosphorylates Rad-51 (Ser-14) and allows it to interact with CK-II. CK-II further phosphorylates and activates Rad-51 at Thr-13 [125]. This double phosphorylation-mediated activation of Rad-51 results in the binding of Rad-51 to the Nijmegen breakage syndrome gene product (NBS). NBS halts the cell cycle in S-phase if DNA errors are present, and can activate the BRCA1/BRCA2 (DNA damage repair proteins) pathway to trigger repair process [123]. Moreover, the Rad-51-NBS complex can facilitate the mobilization and recruitment of Rad-51 to the DNA damage site. Thus, Rad-51 protects cells from genotoxic stress by inducing cellular resistance (Figure 6b). For the first time, these findings have deciphered the role of PLK-1 in the HR-mediated DNA repair mechanism [125].

Thus, the active association of PLK-1 with regulation of checkpoint adaptation evidently proposes that PLK-1 may override the checkpoints in prolonged arrested-cells by activating CDK-1 and CDC25A in response to DNA damage.

## CONCLUSIONS AND FUTURE PROSPECTIVES

Based upon the discussed studies, it is clear that PLK-1 function beyond its’ conventional role in mitosis. Current studies have implicated PLK-1 in the regulation of various events other than mitosis, including ciliogenesis, telomeric-end length, and genomic stability maintenance, and the regulation of p53. Moreover, misexpression of PLK-1 has been shown to be responsible for triggering fatal diseases, such as Alzheimer's disease and tumorigenesis. Taking note of the versatile functional role of PLK-1 in chromosomal segregation, mitosis progression, and organogenesis, its’ participatory role must be studied in other processes, such as neurogenesis, axis patterning, germ layer specification, non-homologous recombination, base and nucleotide excision repair, early birth defects and other neurodegenerative diseases. A detailed analysis of its’ functional role may provide understanding of the neurodegenerative diseases as well as events leading to tumorigenesis. PLK-1 might provide a novel therapeutic target for resolving incurable diseases, such as Alzheimer’s, cancer.

## References

[1] Lee KS, Yuan YL, Kuriyama R, Erikson RL (1995). Plk is an M-phase-specific protein kinase and interacts with a kinesin-like protein, CHO1/MKLP-1. Molecular and cellular biology.

[2] Llamazares S, Moreira A, Tavares A, Girdham C, Spruce BA, Gonzalez C, Karess RE, Glover DM, Sunkel CE (1991). polo encodes a protein kinase homolog required for mitosis in Drosophila. Genes Dev.

[3] Strebhardt K (2010). Multifaceted polo-like kinases: drug targets and antitargets for cancer therapy. Nat Rev Drug Discov.

[4] Sunkel CE, Glover DM (1988). polo, a mitotic mutant of Drosophila displaying abnormal spindle poles. Journal of cell science.

[5] Andrysik Z, Bernstein WZ, Deng L, Myer DL, Li YQ, Tischfield JA, Stambrook PJ, Bahassi el M (2010). The novel mouse Polo-like kinase 5 responds to DNA damage and localizes in the nucleolus. Nucleic Acids Res.

[6] Elia AE, Rellos P, Haire LF, Chao JW, Ivins FJ, Hoepker K, Mohammad D, Cantley LC, Smerdon SJ, Yaffe MB (2003). The molecular basis for phosphodependent substrate targeting and regulation of Plks by the Polo-box domain. Cell.

[7] Lowery DM, Lim D, Yaffe MB (2005). Structure and function of Polo-like kinases. Oncogene.

[8] Li H, Liu XS, Yang X, Wang Y, Turner JR, Liu X (2010). Phosphorylation of CLIP-170 by Plk1 and CK2 promotes timely formation of kinetochore-microtubule attachments. EMBO J.

[9] Li J, Wang R, Schweickert PG, Karki A, Yang Y, Kong Y, Ahmad N, Konieczny SF, Liu X (2016). Plk1 inhibition enhances the efficacy of gemcitabine in human pancreatic cancer. Cell Cycle.

[10] Matsumura S, Toyoshima F, Nishida E (2007). Polo-like kinase 1 facilitates chromosome alignment during prometaphase through BubR1. The Journal of biological chemistry.

[11] Mishima M, Pavicic V, Gruneberg U, Nigg EA, Glotzer M (2004). Cell cycle regulation of central spindle assembly. Nature.

[12] Smith E, Hegarat N, Vesely C, Roseboom I, Larch C, Streicher H, Straatman K, Flynn H, Skehel M, Hirota T, Kuriyama R, Hochegger H (2011). Differential control of Eg5-dependent centrosome separation by Plk1 and Cdk1. EMBO J.

[13] Kumar S, Sharma AR, Sharma G, Chakraborty C, Kim J (2016). PLK-1: Angel or devil for cell cycle progression. Biochimica et biophysica acta.

[14] Kumar S, Kim J (2015). PLK-1 Targeted Inhibitors and Their Potential against Tumorigenesis. BioMed research international.

[15] Spankuch-Schmitt B, Bereiter-Hahn J, Kaufmann M, Strebhardt K (2002). Effect of RNA silencing of polo-like kinase-1 (PLK1) on apoptosis and spindle formation in human cancer cells. J Natl Cancer Inst.

[16] Spankuch-Schmitt B, Wolf G, Solbach C, Loibl S, Knecht R, Stegmuller M, von Minckwitz G, Kaufmann M, Strebhardt K (2002). Downregulation of human polo-like kinase activity by antisense oligonucleotides induces growth inhibition in cancer cells. Oncogene.

[17] Steegmaier M, Hoffmann M, Baum A, Lenart P, Petronczki M, Krssak M, Gurtler U, Garin-Chesa P, Lieb S, Quant J, Grauert M, Adolf GR, Kraut N (2007). BI 2536, a potent and selective inhibitor of polo-like kinase 1, inhibits tumor growth *in vivo*. Curr Biol.

[18] Liu X, Lei M, Erikson RL (2006). Normal cells, but not cancer cells, survive severe Plk1 depletion. Molecular and cellular biology.

[19] Liu XS, Song B, Liu X (2010). The substrates of Plk1, beyond the functions in mitosis. Protein Cell.

[20] Cai Y, Balli D, Ustiyan V, Fulford L, Hiller A, Misetic V, Zhang Y, Paluch AM, Waltz SE, Kasper S, Kalin TV (2013). Foxm1 expression in prostate epithelial cells is essential for prostate carcinogenesis. The Journal of biological chemistry.

[21] Chen YJ, Lin YP, Chow LP, Lee TC (2011). Proteomic identification of Hsp70 as a new Plk1 substrate in arsenic trioxide-induced mitotically arrested cells. Proteomics.

[22] Fairley JA, Mitchell LE, Berg T, Kenneth NS, von Schubert C, Sillje HH, Medema RH, Nigg EA, White RJ (2012). Direct regulation of tRNA and 5S rRNA gene transcription by Polo-like kinase 1. Molecular cell.

[23] Fairley JA, Scott PH, White RJ (2003). TFIIIB is phosphorylated, disrupted and selectively released from tRNA promoters during mitosis *in vivo*. EMBO J.

[24] Meignin C, Davis I (2008). UAP56 RNA helicase is required for axis specification and cytoplasmic mRNA localization in Drosophila. Dev Biol.

[25] Yamazaki T, Fujiwara N, Yukinaga H, Ebisuya M, Shiki T, Kurihara T, Kioka N, Kambe T, Nagao M, Nishida E, Masuda S (2010). The closely related RNA helicases, UAP56 and URH49, preferentially form distinct mRNA export machineries and coordinately regulate mitotic progression. Mol Biol Cell.

[26] Yarm FR (2002). Plk phosphorylation regulates the microtubule-stabilizing protein TCTP. Molecular and cellular biology.

[27] Zhang J, Yuan C, Wu J, Elsayed Z, Fu Z (2015). Polo-like kinase 1-mediated phosphorylation of Forkhead box protein M1b antagonizes its SUMOylation and facilitates its mitotic function. The Journal of biological chemistry.

[28] Bahassi el M (2011). Polo-like kinases and DNA damage checkpoint: beyond the traditional mitotic functions. Exp Biol Med (Maywood).

[29] Hyun SY, Hwang HI, Jang YJ (2014). Polo-like kinase-1 in DNA damage response. BMB reports.

[30] Tavernier N, Panbianco C, Gotta M, Pintard L (2015). Cdk1 plays matchmaker for the Polo-like kinase and its activator SPAT-1/Bora. Cell Cycle.

[31] Macurek L, Lindqvist A, Lim D, Lampson MA, Klompmaker R, Freire R, Clouin C, Taylor SS, Yaffe MB, Medema RH (2008). Polo-like kinase-1 is activated by aurora A to promote checkpoint recovery. Nature.

[32] Seki A, Coppinger JA, Jang CY, Yates JR, Fang G (2008). Bora and the kinase Aurora a cooperatively activate the kinase Plk1 and control mitotic entry. Science.

[33] Abraham RT (2001). Cell cycle checkpoint signaling through the ATM and ATR kinases. Genes Dev.

[34] Park JE, Soung NK, Johmura Y, Kang YH, Liao C, Lee KH, Park CH, Nicklaus MC, Lee KS (2010). Polo-box domain: a versatile mediator of polo-like kinase function. Cell Mol Life Sci.

[35] Laoukili J, Kooistra MR, Bras A, Kauw J, Kerkhoven RM, Morrison A, Clevers H, Medema RH (2005). FoxM1 is required for execution of the mitotic programme and chromosome stability. Nature cell biology.

[36] Cheng XH, Black M, Ustiyan V, Le T, Fulford L, Sridharan A, Medvedovic M, Kalinichenko VV, Whitsett JA, Kalin TV (2014). SPDEF inhibits prostate carcinogenesis by disrupting a positive feedback loop in regulation of the Foxm1 oncogene. PLoS genetics.

[37] Fu Z, Malureanu L, Huang J, Wang W, Li H, van Deursen JM, Tindall DJ, Chen J (2008). Plk1-dependent phosphorylation of FoxM1 regulates a transcriptional programme required for mitotic progression. Nature cell biology.

[38] Zhao R, Shen J, Green MR, MacMorris M, Blumenthal T (2004). Crystal structure of UAP56, a DExD/H-box protein involved in pre-mRNA splicing and mRNA export. Structure.

[39] Xiong F, Lin Y, Han Z, Shi G, Tian L, Wu X, Zeng Q, Zhou Y, Deng J, Chen H (2012). Plk1-mediated phosphorylation of UAP56 regulates the stability of UAP56. Mol Biol Rep.

[40] Zekanowski C, Wojda U (2009). Aneuploidy, chromosomal missegregation, and cell cycle reentry in Alzheimer's disease. Acta neurobiologiae experimentalis.

[41] Selkoe DJ (1999). Translating cell biology into therapeutic advances in Alzheimer's disease. Nature.

[42] Uetake Y, Terada Y, Matuliene J, Kuriyama R (2004). Interaction of Cep135 with a p50 dynactin subunit in mammalian centrosomes. Cell motility and the cytoskeleton.

[43] Vaughan KT, Vallee RB (1995). Cytoplasmic dynein binds dynactin through a direct interaction between the intermediate chains and p150Glued. The Journal of cell biology.

[44] Li H, Liu XS, Yang X, Song B, Wang Y, Liu X (2010). Polo-like kinase 1 phosphorylation of p150Glued facilitates nuclear envelope breakdown during prophase. Proceedings of the National Academy of Sciences of the United States of America.

[45] Song B, Davis K, Liu XS, Lee HG, Smith M, Liu X (2011). Inhibition of Polo-like kinase 1 reduces beta-amyloid-induced neuronal cell death in Alzheimer's disease. Aging (Albany NY).

[46] Seeley ES, Nachury MV (2010). The perennial organelle: assembly and disassembly of the primary cilium. Journal of cell science.

[47] Santos N, Reiter JF (2008). Building it up and taking it down: the regulation of vertebrate ciliogenesis. Developmental dynamics.

[48] Pan J, Snell W (2007). The primary cilium: keeper of the key to cell division. Cell.

[49] Dawe HR, Farr H, Gull K (2007). Centriole/basal body morphogenesis and migration during ciliogenesis in animal cells. Journal of cell science.

[50] Berbari NF, O’Connor AK, Haycraft CJ, Yoder BK (2009). The primary cilium as a complex signaling center. Current biology.

[51] Sharma N, Berbari NF, Yoder BK (2008). Ciliary dysfunction in developmental abnormalities and diseases. Current topics in developmental biology.

[52] Adams M, Smith UM, Logan CV, Johnson CA (2008). Recent advances in the molecular pathology, cell biology and genetics of ciliopathies. Journal of medical genetics.

[53] Fliegauf M, Benzing T, Omran H (2007). When cilia go bad: cilia defects and ciliopathies. Nature reviews Molecular cell biology.

[54] Davenport JR, Yoder BK (2005). An incredible decade for the primary cilium: a look at a once-forgotten organelle. American journal of physiology Renal physiology.

[55] Lee KH, Johmura Y, Yu LR, Park JE, Gao Y, Bang JK, Zhou M, Veenstra TD, Yeon Kim B, Lee KS (2012). Identification of a novel Wnt5a-CK1varepsilon-Dvl2-Plk1-mediated primary cilia disassembly pathway. The EMBO journal.

[56] Nourry C, Maksumova L, Pang M, Liu X, Wang T (2004). Direct interaction between Smad3, APC10, CDH1 and HEF1 in proteasomal degradation of HEF1. BMC cell biology.

[57] Liu X, Elia AE, Law SF, Golemis EA, Farley J, Wang T (2000). A novel ability of Smad3 to regulate proteasomal degradation of a Cas family member HEF1. The EMBO journal.

[58] Li J, Karki A, Hodges KB, Ahmad N, Zoubeidi A, Strebhardt K, Ratliff TL, Konieczny SF, Liu X (2015). Cotargeting Polo-Like Kinase 1 and the Wnt/beta-Catenin Signaling Pathway in Castration-Resistant Prostate Cancer. Molecular and cellular biology.

[59] Watrin E, Legagneux V (2003). Introduction to chromosome dynamics in mitosis. Biology of the cell / under the auspices of the European Cell Biology Organization.

[60] St-Pierre J, Douziech M, Bazile F, Pascariu M, Bonneil E, Sauve V, Ratsima H, D’Amours D (2009). Polo kinase regulates mitotic chromosome condensation by hyperactivation of condensin DNA supercoiling activity. Molecular cell.

[61] Kimura K, Hirano M, Kobayashi R, Hirano T (1998). Phosphorylation and activation of 13S condensin by Cdc2 *in vitro*. Science.

[62] Kurasawa Y, Yu-Lee LY (2010). PICH and cotargeted Plk1 coordinately maintain prometaphase chromosome arm architecture. Molecular biology of the cell.

[63] Durrieu F, Samejima K, Fortune JM, Kandels-Lewis S, Osheroff N, Earnshaw WC (2000). DNA topoisomerase IIalpha interacts with CAD nuclease and is involved in chromatin condensation during apoptotic execution. Current biology.

[64] Ishida R, Sato M, Narita T, Utsumi KR, Nishimoto T, Morita T, Nagata H, Andoh T (1994). Inhibition of DNA topoisomerase II by ICRF-193 induces polyploidization by uncoupling chromosome dynamics from other cell cycle events. The Journal of cell biology.

[65] Gasser SM, Walter R, Dang Q, Cardenas ME (1992). Topoisomerase II: its functions and phosphorylation. Antonie van Leeuwenhoek.

[66] (2013). A chromatin remodeling complex regulates topoisomerase IIalpha function. Cancer discovery.

[67] Dykhuizen EC, Hargreaves DC, Miller EL, Cui K, Korshunov A, Kool M, Pfister S, Cho YJ, Zhao K, Crabtree GR (2013). BAF complexes facilitate decatenation of DNA by topoisomerase IIalpha. Nature.

[68] Li H, Wang Y, Liu X (2008). Plk1-dependent phosphorylation regulates functions of DNA topoisomerase IIalpha in cell cycle progression. The Journal of biological chemistry.

[69] Garton M, Laughton C (2013). A comprehensive model for the recognition of human telomeres by TRF1. Journal of molecular biology.

[70] Wu ZQ, Yang X, Weber G, Liu X (2008). Plk1 phosphorylation of TRF1 is essential for its binding to telomeres. The Journal of biological chemistry.

[71] Liu X, Erikson RL (2003). Polo-like kinase (Plk)1 depletion induces apoptosis in cancer cells. Proceedings of the National Academy of Sciences of the United States of America.

[72] Ando K, Ozaki T, Yamamoto H, Furuya K, Hosoda M, Hayashi S, Fukuzawa M, Nakagawara A (2004). Polo-like kinase 1 (Plk1) inhibits p53 function by physical interaction and phosphorylation. The Journal of biological chemistry.

[73] Liu XS, Li H, Song B, Liu X (2010). Polo-like kinase 1 phosphorylation of G2 and S-phase-expressed 1 protein is essential for p53 inactivation during G2 checkpoint recovery. EMBO Rep.

[74] Weger S, Hammer E, Heilbronn R (2005). Topors acts as a SUMO-1 E3 ligase for p53 *in vitro* and *in vivo*. FEBS Lett.

[75] Yang X, Li H, Deng A, Liu X (2010). Plk1 phosphorylation of Topors is involved in its degradation. Mol Biol Rep.

[76] Incassati A, Patel D, McCance DJ (2006). Induction of tetraploidy through loss of p53 and upregulation of Plk1 by human papillomavirus type-16 E6. Oncogene.

[77] Patel D, Incassati A, Wang N, McCance DJ (2004). Human papillomavirus type 16 E6 and E7 cause polyploidy in human keratinocytes and up-regulation of G2-M-phase proteins. Cancer research.

[78] Zhou R, Wen H, Ao SZ (1999). Identification of a novel gene encoding a p53-associated protein. Gene.

[79] Pungaliya P, Kulkarni D, Park HJ, Marshall H, Zheng H, Lackland H, Saleem A, Rubin EH (2007). TOPORS functions as a SUMO-1 E3 ligase for chromatin-modifying proteins. Journal of proteome research.

[80] Haluska P, Saleem A, Rasheed Z, Ahmed F, Su EW, Liu LF, Rubin EH (1999). Interaction between human topoisomerase I and a novel RING finger/arginine-serine protein. Nucleic acids research.

[81] Saleem A, Dutta J, Malegaonkar D, Rasheed F, Rasheed Z, Rajendra R, Marshall H, Luo M, Li H, Rubin EH (2004). The topoisomerase I- and p53-binding protein topors is differentially expressed in normal and malignant human tissues and may function as a tumor suppressor. Oncogene.

[82] Lin L, Ozaki T, Takada Y, Kageyama H, Nakamura Y, Hata A, Zhang JH, Simonds WF, Nakagawara A, Koseki H (2005). topors, a p53 and topoisomerase I-binding RING finger protein, is a coactivator of p53 in growth suppression induced by DNA damage. Oncogene.

[83] Hammer E, Heilbronn R, Weger S (2007). The E3 ligase Topors induces the accumulation of polysumoylated forms of DNA topoisomerase I *in vitro* and *in vivo*. FEBS letters.

[84] Guan B, Pungaliya P, Li X, Uquillas C, Mutton LN, Rubin EH, Bieberich CJ (2008). Ubiquitination by TOPORS regulates the prostate tumor suppressor NKX3.1. The Journal of biological chemistry.

[85] Rajendra R, Malegaonkar D, Pungaliya P, Marshall H, Rasheed Z, Brownell J, Liu LF, Lutzker S, Saleem A, Rubin EH (2004). Topors functions as an E3 ubiquitin ligase with specific E2 enzymes and ubiquitinates p53. The Journal of biological chemistry.

[86] Liu XM, Yang FF, Yuan YF, Zhai R, Huo LJ (2013). SUMOylation of mouse p53b by SUMO-1 promotes its pro-apoptotic function in ovarian granulosa cells. PloS one.

[87] Utrera R, Collavin L, Lazarevic D, Delia D, Schneider C (1998). A novel p53-inducible gene coding for a microtubule-localized protein with G2-phase-specific expression. EMBO J.

[88] Monte M, Benetti R, Collavin L, Marchionni L, Del Sal G, Schneider C (2004). hGTSE-1 expression stimulates cytoplasmic localization of p53. The Journal of biological chemistry.

[89] Monte M, Benetti R, Buscemi G, Sandy P, Del Sal G, Schneider C (2003). The cell cycle-regulated protein human GTSE-1 controls DNA damage-induced apoptosis by affecting p53 function. The Journal of biological chemistry.

[90] Holohan C, Van Schaeybroeck S, Longley DB, Johnston PG (2013). Cancer drug resistance: an evolving paradigm. Nature reviews Cancer.

[91] Zahreddine H, Borden KL (2013). Mechanisms and insights into drug resistance in cancer. Frontiers in pharmacology.

[92] Song B, Liu XS, Davis K, Liu X (2011). Plk1 phosphorylation of Orc2 promotes DNA replication under conditions of stress. Molecular and cellular biology.

[93] Plunkett W, Huang P, Xu YZ, Heinemann V, Grunewald R, Gandhi V (1995). Gemcitabine: metabolism, mechanisms of action, and self-potentiation. Seminars in oncology.

[94] Shao C, Ahmad N, Hodges K, Kuang S, Ratliff T, Liu X (2015). Inhibition of polo-like kinase 1 (Plk1) enhances the antineoplastic activity of metformin in prostate cancer. The Journal of biological chemistry.

[95] Viollet B, Guigas B, Sanz Garcia N, Leclerc J, Foretz M, Andreelli F (2012). Cellular and molecular mechanisms of metformin: an overview. Clin Sci (Lond).

[96] Zhang Z, Hou X, Shao C, Li J, Cheng JX, Kuang S, Ahmad N, Ratliff T, Liu X (2014). Plk1 inhibition enhances the efficacy of androgen signaling blockade in castration-resistant prostate cancer. Cancer research.

[97] Harper JW, Elledge SJ (2007). The DNA damage response: ten years after. Molecular cell.

[98] Bartek J, Lukas J (2007). DNA damage checkpoints: from initiation to recovery or adaptation. Curr Opin Cell Biol.

[99] Strebhardt K, Ullrich A (2006). Targeting polo-like kinase 1 for cancer therapy. Nature reviews Cancer.

[100] Inoue D, Sagata N (2005). The Polo-like kinase Plx1 interacts with and inhibits Myt1 after fertilization of Xenopus eggs. EMBO J.

[101] Roshak AK, Capper EA, Imburgia C, Fornwald J, Scott G, Marshall LA (2000). The human polo-like kinase, PLK, regulates cdc2/cyclin B through phosphorylation and activation of the cdc25C phosphatase. Cell Signal.

[102] Watanabe N, Arai H, Iwasaki J, Shiina M, Ogata K, Hunter T, Osada H (2005). Cyclin-dependent kinase (CDK) phosphorylation destabilizes somatic Wee1 via multiple pathways. Proceedings of the National Academy of Sciences of the United States of America.

[103] Watanabe N, Arai H, Nishihara Y, Taniguchi M, Hunter T, Osada H (2004). M-phase kinases induce phospho-dependent ubiquitination of somatic Wee1 by SCFbeta-TrCP. Proceedings of the National Academy of Sciences of the United States of America.

[104] Matthew EM, Yen TJ, Dicker DT, Dorsey JF, Yang W, Navaraj A, El-Deiry WS (2007). Replication stress, defective S-phase checkpoint and increased death in Plk2-deficient human cancer cells. Cell Cycle.

[105] Falck J, Mailand N, Syljuasen RG, Bartek J, Lukas J (2001). The ATM-Chk2-Cdc25A checkpoint pathway guards against radioresistant DNA synthesis. Nature.

[106] Kumagai A, Dunphy WG (2003). Repeated phosphopeptide motifs in Claspin mediate the regulated binding of Chk1. Nature cell biology.

[107] Mailand N, Bekker-Jensen S, Bartek J, Lukas J (2006). Destruction of Claspin by SCFbetaTrCP restrains Chk1 activation and facilitates recovery from genotoxic stress. Molecular cell.

[108] Yoo HY, Kumagai A, Shevchenko A, Dunphy WG (2004). Adaptation of a DNA replication checkpoint response depends upon inactivation of Claspin by the Polo-like kinase. Cell.

[109] Kops GJ, Weaver BA, Cleveland DW (2005). On the road to cancer: aneuploidy and the mitotic checkpoint. Nature reviews Cancer.

[110] Taylor WR, Stark GR (2001). Regulation of the G2/M transition by p53. Oncogene.

[111] van Vugt MA, Gardino AK, Linding R, Ostheimer GJ, Reinhardt HC, Ong SE, Tan CS, Miao H, Keezer SM, Li J, Pawson T, Lewis TA, Carr SA (2010). A mitotic phosphorylation feedback network connects Cdk1, Plk1, 53BP1, and Chk2 to inactivate the G(2)/M DNA damage checkpoint. PLoS Biol.

[112] Goldar S, Khaniani MS, Derakhshan SM, Baradaran B (2015). Molecular mechanisms of apoptosis and roles in cancer development and treatment. Asian Pacific journal of cancer prevention.

[113] Plati J, Bucur O, Khosravi-Far R (2011). Apoptotic cell signaling in cancer progression and therapy. Integrative biology.

[114] Fan Y, Zheng S, Xu ZF, Ding JY (2005). Apoptosis induction with polo-like kinase-1 antisense phosphorothioate oligodeoxynucleotide of colon cancer cell line SW480. World journal of gastroenterology.

[115] Koida N, Ozaki T, Yamamoto H, Ono S, Koda T, Ando K, Okoshi R, Kamijo T, Omura K, Nakagawara A (2008). Inhibitory role of Plk1 in the regulation of p73-dependent apoptosis through physical interaction and phosphorylation. The Journal of biological chemistry.

[116] Soond SM, Barry SP, Melino G, Knight RA, Latchman DS, Stephanou A (2008). p73-mediated transcriptional activity is negatively regulated by polo-like kinase 1. Cell Cycle.

[117] Kreis NN, Sommer K, Sanhaji M, Kramer A, Matthess Y, Kaufmann M, Strebhardt K, Yuan J (2009). Long-term downregulation of Polo-like kinase 1 increases the cyclin-dependent kinase inhibitor p21(WAF1/CIP1). Cell Cycle.

[118] Galgoczy DJ, Toczyski DP (2001). Checkpoint adaptation precedes spontaneous and damage-induced genomic instability in yeast. Molecular and cellular biology.

[119] Sandell LL, Zakian VA (1993). Loss of a yeast telomere: arrest, recovery, and chromosome loss. Cell.

[120] Amin MA, Itoh G, Iemura K, Ikeda M, Tanaka K (2014). CLIP-170 recruits PLK1 to kinetochores during early mitosis for chromosome alignment. Journal of cell science.

[121] Donnianni RA, Ferrari M, Lazzaro F, Clerici M, Tamilselvan Nachimuthu B, Plevani P, Muzi-Falconi M, Pellicioli A (2010). Elevated levels of the polo kinase Cdc5 override the Mec1/ATR checkpoint in budding yeast by acting at different steps of the signaling pathway. PLoS genetics.

[122] Syljuasen RG, Jensen S, Bartek J, Lukas J (2006). Adaptation to the ionizing radiation-induced G2 checkpoint occurs in human cells and depends on checkpoint kinase 1 and Polo-like kinase 1 kinases. Cancer research.

[123] Falck J, Forment JV, Coates J, Mistrik M, Lukas J, Bartek J, Jackson SP (2012). CDK targeting of NBS1 promotes DNA-end resection, replication restart and homologous recombination. EMBO Rep.

[124] Lee SE, Pellicioli A, Malkova A, Foiani M, Haber JE (2001). The Saccharomyces recombination protein Tid1p is required for adaptation from G2/M arrest induced by a double-strand break. Curr Biol.

[125] Yata K, Lloyd J, Maslen S, Bleuyard JY, Skehel M, Smerdon SJ, Esashi F (2012). Plk1 and CK2 act in concert to regulate Rad51 during DNA double strand break repair. Molecular cell.

[126] Tamura Y, Simizu S, Muroi M, Takagi S, Kawatani M, Watanabe N, Osada H (2009). Polo-like kinase 1 phosphorylates and regulates Bcl-x(L) during pironetin-induced apoptosis. Oncogene.

[127] Arai T, Haze K, Iimura-Morita Y, Machida T, Iida M, Tanaka K, Komatani H (2008). Identification of beta-catenin as a novel substrate of Polo-like kinase 1. Cell Cycle.

[128] Lin HR, Ting NS, Qin J, Lee WH (2003). M phase-specific phosphorylation of BRCA2 by Polo-like kinase 1 correlates with the dissociation of the BRCA2-P/CAF complex. The Journal of biological chemistry.

[129] Zhang H, Shi X, Paddon H, Hampong M, Dai W, Pelech S (2004). B23/nucleophosmin serine 4 phosphorylation mediates mitotic functions of polo-like kinase 1. The Journal of biological chemistry.

[130] Qi W, Tang Z, Yu H (2006). Phosphorylation- and polo-box-dependent binding of Plk1 to Bub1 is required for the kinetochore localization of Plk1. Mol Biol Cell.

[131] Elowe S, Hummer S, Uldschmid A, Li X, Nigg EA (2007). Tension-sensitive Plk1 phosphorylation on BubR1 regulates the stability of kinetochore microtubule interactions. Genes Dev.

[132] Toyoshima-Morimoto F, Taniguchi E, Nishida E (2002). Plk1 promotes nuclear translocation of human Cdc25C during prophase. EMBO Rep.

[133] Fabbro M, Zhou BB, Takahashi M, Sarcevic B, Lal P, Graham ME, Gabrielli BG, Robinson PJ, Nigg EA, Ono Y, Khanna KK (2005). Cdk1/Erk2- and Plk1-dependent phosphorylation of a centrosome protein, Cep55, is required for its recruitment to midbody and cytokinesis. Dev Cell.

[134] Guarguaglini G, Duncan PI, Stierhof YD, Holmstrom T, Duensing S, Nigg EA (2005). The forkhead-associated domain protein Cep170 interacts with Polo-like kinase 1 and serves as a marker for mature centrioles. Mol Biol Cell.

[135] Toyoshima-Morimoto F, Taniguchi E, Shinya N, Iwamatsu A, Nishida E (2001). Polo-like kinase 1 phosphorylates cyclin B1 and targets it to the nucleus during prophase. Nature.

[136] Jackman M, Lindon C, Nigg EA, Pines J (2003). Active cyclin B1-Cdk1 first appears on centrosomes in prophase. Nature cell biology.

[137] Moshe Y, Boulaire J, Pagano M, Hershko A (2004). Role of Polo-like kinase in the degradation of early mitotic inhibitor 1, a regulator of the anaphase promoting complex/cyclosome. Proceedings of the National Academy of Sciences of the United States of America.

[138] Preisinger C, Korner R, Wind M, Lehmann WD, Kopajtich R, Barr FA (2005). Plk1 docking to GRASP65 phosphorylated by Cdk1 suggests a mechanism for Golgi checkpoint signalling. EMBO J.

[139] Wu ZQ, Liu X (2008). Role for Plk1 phosphorylation of Hbo1 in regulation of replication licensing.

[140] Wolfe BA, Takaki T, Petronczki M, Glotzer M (2009). Polo-like kinase 1 directs assembly of the HsCyk-4 RhoGAP/Ect2 RhoGEF complex to initiate cleavage furrow formation. PLoS Biol.

[141] Burkard ME, Maciejowski J, Rodriguez-Bravo V, Repka M, Lowery DM, Clauser KR, Zhang C, Shokat KM, Carr SA, Yaffe MB, Jallepalli PV (2009). Plk1 self-organization and priming phosphorylation of HsCYK-4 at the spindle midzone regulate the onset of division in human cells. PLoS Biol.

[142] Kim SA, Yoon JH, Lee SH, Ahn SG (2005). Polo-like kinase 1 phosphorylates heat shock transcription factor 1 and mediates its nuclear translocation during heat stress. The Journal of biological chemistry.

[143] Lee YJ, Kim EH, Lee JS, Jeoung D, Bae S, Kwon SH, Lee YS (2008). HSF1 as a mitotic regulator: phosphorylation of HSF1 by Plk1 is essential for mitotic progression. Cancer research.

[144] Higashimoto T, Chan N, Lee YK, Zandi E (2008). Regulation of I(kappa)B kinase complex by phosphorylation of (gamma)-binding domain of I(kappa)B kinase (beta) by Polo-like kinase 1. The Journal of biological chemistry.

[145] Chen L, Li Z, Ahmad N, Liu X (2015). Plk1 phosphorylation of IRS2 prevents premature mitotic exit via AKT inactivation. Biochemistry.

[146] Jang CY, Coppinger JA, Seki A, Yates JR, Fang G (2009). Plk1 and Aurora A regulate the depolymerase activity and the cellular localization of Kif2a. Journal of cell science.

[147] Hood EA, Kettenbach AN, Gerber SA, Compton DA (2012). Plk1 regulates the kinesin-13 protein Kif2b to promote faithful chromosome segregation. Mol Biol Cell.

[148] Oshimori N, Ohsugi M, Yamamoto T (2006). The Plk1 target Kizuna stabilizes mitotic centrosomes to ensure spindle bipolarity. Nature cell biology.

[149] Liu X, Zhou T, Kuriyama R, Erikson RL (2004). Molecular interactions of Polo-like-kinase 1 with the mitotic kinesin-like protein CHO1/MKLP-1. Journal of cell science.

[150] Neef R, Preisinger C, Sutcliffe J, Kopajtich R, Nigg EA, Mayer TU, Barr FA (2003). Phosphorylation of mitotic kinesin-like protein 2 by polo-like kinase 1 is required for cytokinesis. J Cell Biol.

[151] Nakajima H, Toyoshima-Morimoto F, Taniguchi E, Nishida E (2003). Identification of a consensus motif for Plk (Polo-like kinase) phosphorylation reveals Myt1 as a Plk1 substrate. The Journal of biological chemistry.

[152] Asiedu M, Wu D, Matsumura F, Wei Q (2008). Phosphorylation of MyoGEF on Thr-574 by Plk1 promotes MyoGEF localization to the central spindle. The Journal of biological chemistry.

[153] Zhang X, Chen Q, Feng J, Hou J, Yang F, Liu J, Jiang Q, Zhang C (2009). Sequential phosphorylation of Nedd1 by Cdk1 and Plk1 is required for targeting of the gammaTuRC to the centrosome. Journal of cell science.

[154] Casenghi M, Meraldi P, Weinhart U, Duncan PI, Korner R, Nigg EA (2003). Polo-like kinase 1 regulates Nlp, a centrosome protein involved in microtubule nucleation. Dev Cell.

[155] Zhou T, Aumais JP, Liu X, Yu-Lee LY, Erikson RL (2003). A role for Plk1 phosphorylation of NudC in cytokinesis. Dev Cell.

[156] Kachaner D, Filipe J, Laplantine E, Bauch A, Bennett KL, Superti-Furga G, Israel A, Weil R (2012). Plk1-dependent phosphorylation of optineurin provides a negative feedback mechanism for mitotic progression. Molecular cell.

[157] Hansen DV, Loktev AV, Ban KH, Jackson PK (2004). Plk1 regulates activation of the anaphase promoting complex by phosphorylating and triggering SCFbetaTrCP-dependent destruction of the APC Inhibitor Emi1. Mol Biol Cell.

[158] Baumann C, Korner R, Hofmann K, Nigg EA (2007). PICH, a centromere-associated SNF2 family ATPase, is regulated by Plk1 and required for the spindle checkpoint. Cell.

[159] Eckerdt F, Yuan J, Saxena K, Martin B, Kappel S, Lindenau C, Kramer A, Naumann S, Daum S, Fischer G, Dikic I, Kaufmann M, Strebhardt K (2005). Polo-like kinase 1-mediated phosphorylation stabilizes Pin1 by inhibiting its ubiquitination in human cells. The Journal of biological chemistry.

[160] Pahlavan G, Polanski Z, Kalab P, Golsteyn R, Nigg EA, Maro B (2000). Characterization of polo-like kinase 1 during meiotic maturation of the mouse oocyte. Dev Biol.

[161] Li Z, Li J, Bi P, Lu Y, Burcham G, Elzey BD, Ratliff T, Konieczny SF, Ahmad N, Kuang S, Liu X (2014). Plk1 phosphorylation of PTEN causes a tumor-promoting metabolic state. Molecular and cellular biology.

[162] Feng Y, Yuan JH, Maloid SC, Fisher R, Copeland TD, Longo DL, Conrads TP, Veenstra TD, Ferris A, Hughes S, Dimitrov DS, Ferris DK (2006). Polo-like kinase 1-mediated phosphorylation of the GTP-binding protein Ran is important for bipolar spindle formation. Biochem Biophys Res Commun.

[163] Shao T, Liu X (2015). Identification of rictor as a novel substrate of Polo-like kinase 1. Cell Cycle.

[164] Lowery DM, Clauser KR, Hjerrild M, Lim D, Alexander J, Kishi K, Ong SE, Gammeltoft S, Carr SA, Yaffe MB (2007). Proteomic screen defines the Polo-box domain interactome and identifies Rock2 as a Plk1 substrate. EMBO J.

[165] Liu XS, Song B, Tang J, Liu W, Kuang S, Liu X (2012). Plk1 phosphorylates Sgt1 at the kinetochores to promote timely kinetochore-microtubule attachment. Molecular and cellular biology.

[166] Hudson JW, Kozarova A, Cheung P, Macmillan JC, Swallow CJ, Cross JC, Dennis JW (2001). Late mitotic failure in mice lacking Sak, a polo-like kinase. Curr Biol.

[167] Fleckner J, Zhang M, Valcarcel J, Green MR (1997). U2AF65 recruits a novel human DEAD box protein required for the U2 snRNP-branchpoint interaction. Genes Dev.

[168] Yamaguchi T, Goto H, Yokoyama T, Sillje H, Hanisch A, Uldschmid A, Takai Y, Oguri T, Nigg EA, Inagaki M (2005). Phosphorylation by Cdk1 induces Plk1-mediated vimentin phosphorylation during mitosis. J Cell Biol.

